# How compliance with behavioural measures during the initial phase of a pandemic develops over time: A longitudinal COVID‐19 study

**DOI:** 10.1111/bjso.12572

**Published:** 2022-10-10

**Authors:** Monique Chambon, Jonas Dalege, Denny Borsboom, Lourens J. Waldorp, Han L. J. van der Maas, Frenk van Harreveld

**Affiliations:** ^1^ National Institute for Public Health and the Environment (RIVM) Bilthoven The Netherlands; ^2^ Department of Psychology University of Amsterdam Amsterdam The Netherlands; ^3^ Santa Fe Institute Santa Fe New Mexico USA

**Keywords:** broad attitude networks, compliance with behavioural measures, COVID‐19 pandemic, longitudinal, within‐person temporal effects

## Abstract

In this longitudinal research, we adopt a complexity approach to examine the temporal dynamics of variables related to compliance with behavioural measures during the COVID‐19 pandemic. Dutch participants (*N* = 2399) completed surveys with COVID‐19‐related variables five times over a period of 10 weeks (23 April–30 June 2020). With these data, we estimated within‐person COVID‐19 attitude networks containing a broad set of psychological variables and their relations. These networks display variables' predictive effects over time between measurements and contemporaneous effects during measurements. Results show (1) bidirectional effects between multiple variables relevant for compliance, forming potential feedback loops, and (2) a positive reinforcing structure between compliance, support for behavioural measures, involvement in the pandemic and vaccination intention. These results can explain why levels of these variables decreased throughout the course of the study. The reinforcing structure points towards potentially amplifying effects of interventions on these variables and might inform processes of polarization. We conclude that adopting a complexity approach might contribute to understanding protective behaviour in the initial phase of pandemics by combining different theoretical models and modelling bidirectional effects between variables. Future research could build upon this research by studying causality with interventions and including additional variables in the networks.

## BACKGROUND

During the initial phase of a pandemic, a vaccine is often absent and compliance with behavioural measures such as social distancing and isolation are often considered of pivotal importance to curtail the spread of the virus. Research during the COVID‐19 pandemic shows that the majority of people reported compliance with behavioural measures but also indicates ample room for improvement (Hensel et al., 2022). Thus, greater insight into determinants of compliance is crucially important for improving our ability to understand, predict and improve compliance during epidemic outbreaks (Betsch et al., 2020).

The literature on compliance with behavioural guidelines during the COVID‐19 pandemic is already immense, with numerous studies pointing towards factors contributing to compliance or the lack thereof, from a range of theoretical perspectives, such as the Theory of Planned Behaviour (Ajzen, 1991; Gibson et al., 2021), the Health Belief Model (Clark et al., 2020; Rosenstock, 1974) and Moral Foundations Theory (Chan, 2021; Graham et al., 2013). This body of work has generated important insight into the psychological underpinnings of compliance in the context of the COVID‐19 pandemic, but theoretical frameworks like the ones just mentioned are inherently restrictive because of the limited number of variables they focus on. An influential perspective on compliance during pandemics that is less restrictive is that by Bish and Michie (2010), who provide an extensive review of the literature on this topic and discuss a wide range of determinants that play a role in predicting health behaviour during a pandemic. As such, most empirical work on this topic takes the perspective of a particular model and the specific elements in it, while the influential perspective by Bish and Michie takes a broader perspective but is theoretical in nature.

Our current aim is to deepen our understanding of compliance with behavioural measures during the initial phase of a pandemic by taking a complexity approach. We do so in accordance with the conceptual frameworks provided by Bish and Michie (2010). We add to this existing literature by conducting empirical research that includes not only the constructs of these conceptual frameworks but also additional variables that more recent research showed to be relevant in the context of pandemics. Moreover, we employ a longitudinal design that provides insight into how relations between variables develop over time and thus indications of causal effects. Such longitudinal designs seem to be most lacking in the literature on pandemics thus far (Bish & Michie, 2010).

## Psychological variables related to compliance during pandemics

Bish and Michie (2010) propose frameworks on health behaviour during a pandemic with different attitudinal and demographic determinants depending on the type of protective behaviour. In our current research, we focussed on preventive behaviours (e.g. hygiene behaviours and vaccination) and avoidant behaviours (e.g. social distancing and working from home) since these resonate with the behavioural measures as advised by Dutch authorities during the initial phase of the pandemic. According to Bish and Michie (2010), attitudinal variables that are associated with preventive and avoidant behaviours include perceived severity, susceptibility, costs, efficacy and self‐efficacy but also social norms, cues to action, knowledge, trust in authorities and state anxiety. Moreover, they argue most of these variables are covered by three well‐known models within psychology that have been used to explain relevant behaviour, namely the Theory of Planned Behaviour (TPB; Ajzen, 1991), the Health Belief Model (HBM; Rosenstock, 1974) and the Protection Motivation Theory (PMT; Rogers, 1975). The TPB predicts behaviour through intention, with the intention being influenced by attitudes, behavioural control and behavioural norms. The HBM explains health‐related behaviour through the perception of one's susceptibility and severity of the disease, benefits and barriers of the behaviour, self‐efficacy and cues to action. PMT seeks to understand behaviour through threat appraisal (i.e. severity of and vulnerability to the disease) and coping appraisal (i.e. response and self‐efficacy). Research during the COVID‐19 pandemic also associates components of these models with compliance with behavioural measures, namely attitudes (Bogg & Milad, 2020), perceived control and severity (Li et al., 2020), risk perception (Schneider et al., 2021), social norms (Tunçgenç et al., 2021) and self‐efficacy (Shahnazi et al., 2020).

Bish and Michie (2010) argue that various psychological factors not included in these models are also relevant in the context of pandemics, such as knowledge about the disease, trust in authorities and state anxiety. The latter suggests that adverse effects of the pandemic on people's mental health should also be considered when aiming to understand behaviour in the context of pandemics. Recent research during the COVID‐19 pandemic indeed associates compliance with behavioural measures with mental health, namely fear of the virus (Harper et al., 2021) and stress (Lieberoth et al., 2021). Additionally, potential consequences of behavioural measures such as changes in lifestyle and loneliness are associated with adverse mental and physical health effects (Blom et al., 2021; Leigh‐Hunt et al., 2017). These findings suggest it is important to take changes in (mental) health into account when investigating compliance during a pandemic.

Thus, research points towards a wide range of interrelated variables relevant to health behaviour during a pandemic. Chambon, Dalege, Elberse, et al. (2022) corroborated this in the context of the COVID‐19 pandemic by adopting a complex psychological systems approach to provide an overview of variables related to preventive behaviours in a study conducted in both the United Kingdom and the Netherlands. This resulted in a descriptive account of how these variables are interrelated and insight into differences between the countries. The study could not provide insight into the directions of relations between variables and their temporal effects due to its cross‐sectional design (Chambon, Dalege, Elberse, et al., 2022).

Prior research suggests that temporal effects are an important subject of investigation because psychological factors associated with compliance with behavioural measures during pandemics can show substantive changes over time (Ibuka et al., 2010; van der Weerd et al., 2011). Likewise, research during the COVID‐19 pandemic showed fluctuations in risk perception (Schneider et al., 2021), psychological distress (Daly & Robinson, 2021) and well‐being (Wang et al., 2021). Such temporal changes in psychological factors associated with compliance highlight the importance of longitudinal research into compliance during pandemics. Accordingly, the current research contributes to the existing literature by examining the temporal effects of variables and their interrelations. We focus on variables that can be expected to fluctuate during the initial phase of a pandemic and not relatively stable variables such as demographics, personality traits and political preferences. We recognize that such variables can be important for compliance as well and may moderate the relations between variables included in this study, but their effects fall outside of the scope of the present research.

To summarize, prior research showed that a broad set of variables can affect compliance with behavioural measures during pandemics. Also, there is a gap in the literature regarding longitudinal research that provides insight into the temporal effects of variables relevant to protective behaviours during a pandemic. Consequently, parsimonious theoretical models might not provide sufficient insight into the complexity of compliance during pandemics. Nevertheless, most research into compliance focussed on a rather limited set of variables examined in a cross‐sectional research design. The current research therefore adopts a complexity approach in which a more diverse set of variables was included and employs a longitudinal research design. Importantly, the aim of this research is not to present a comprehensive overview or an exhaustive set of variables relevant for compliance but to demonstrate that compliance during pandemics can be explained by a broad set of variables that extends beyond the traditional models (i.e. complexity approach) and to provide insight into directions of effects between these variables (i.e. temporal effects).

## Complexity approach

We utilize a complexity approach to gain insight into how psychological variables organize and interact over time. A promising means to this goal is psychological network analysis, which (1) enables exploration of data with many variables of different types, (2) provides insight into patterns of unique relations that remain after controlling for effects of other variables (i.e. pairwise conditional dependencies) and (3) presents these statistical associations in powerful visualizations (Borsboom et al., 2021). A theoretically underpinned model of attitudes that adopts a complexity approach through network analysis is the Causal Attitude Network (CAN) model (Dalege et al., 2016). This model conceptualizes attitudes as complex systems of interacting cognitive, affective and behavioural factors. This systemic approach models properties of attitude dynamics, including their relation to behaviour. Because this approach naturally integrates many different factors relevant to behaviour, we adopt an extension of the CAN model to improve our understanding of compliance behaviour.

**FIGURE 1:**
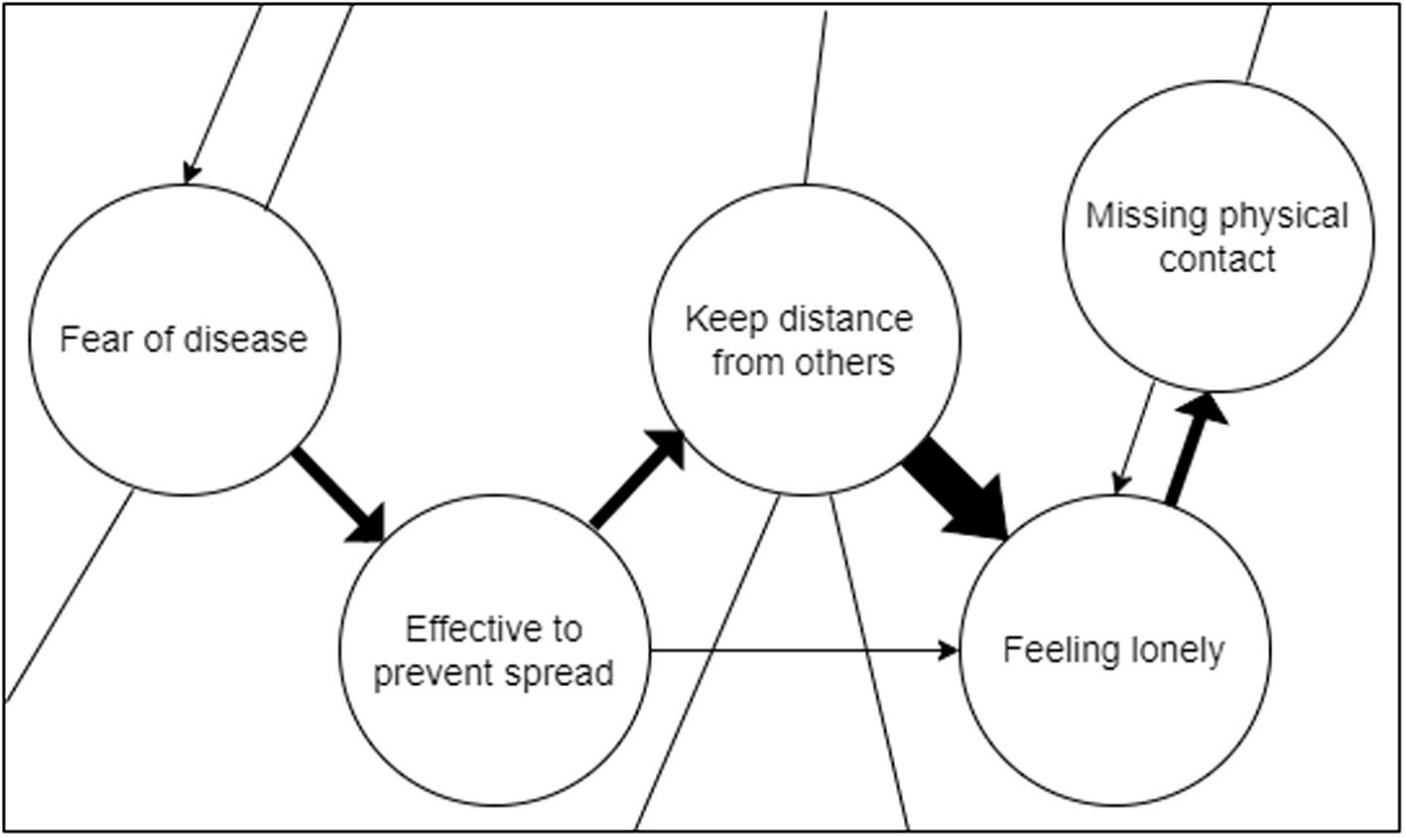
Simplified and hypothetical attitude network of the attitude towards physical distancing. Variables are displayed as nodes (circles) and linear relations between variables as edges (lines between circles). The arrows indicate predictive effects between nodes from one measurement to the next. The network consists of a behavioural element (‘Keep distance from others’), a cognitive evaluation (‘Physical distance is effective to prevent spread’) and three affective evaluations (‘Fear of disease’, ‘Feeling lonely’ and ‘Missing physical contact’). The edge width indicates the strength of the relations. In this example, feeling lonely is more strongly affected by keeping distance from others than by perceiving physical distance as effective to prevent the spread, as indicated by the different width of these edges in the network. Furthermore, relations can be unidirectional (e.g. between keeping distance from others and feeling lonely) or bidirectional (e.g. between feeling lonely and missing physical contact). Bidirectional effects can consist of edges with different strengths

Attitude networks in the CAN model consist of nodes that represent cognitive, affective and behavioural components of attitudes. Links between nodes represent interactions between these components. These links, also known as edges, represent linear relations between nodes and can be positive, indicating an excitatory relation, or negative, indicating an inhibitory relation. The strength of interactions between nodes can vary, resulting in a weighted network. In the CAN model, the overall state of the system (i.e. the pattern of feelings, thoughts and behaviours that characterize a person) arises out of these interactions between attitude components (Dalege et al., 2016). Figure 1 shows a simplified and hypothetical network of the attitude towards physical distancing. Through patterns of interaction, some node pairs will be more strongly aligned than others. These patterns can be identified using partial correlations, where the relation between two given nodes is conditional on all other nodes in the network (see Epskamp, 2020a; van Borkulo et al., 2014). Thus, via statistical analyses of data, empirical estimates of attitude network models can be obtained (Dalege et al., 2017). Representations of these models can yield important information to advance our understanding of behavioural variables. For example, the centrality of specific nodes may provide information on the relative importance of different nodes in the network organization, that is, nodes central to the network have more and stronger relations with other nodes. Ideally, network models are estimated on longitudinal data, as such data can harbour important clues about the causal organization of the network. This results in network models that include directions of effects between variables (see Figure 1).

To summarize, the present research addresses gaps in the literature regarding complexity and temporal effects by shedding light on temporal dynamics of the system of psychological factors (i.e. broad attitudes networks) related to compliance with behavioural measures during the COVID‐19 pandemic. Our means in doing so is conducting network analysis with longitudinal data gathered during the pandemic: participants completed a survey with COVID‐19‐related variables five times over a period of approximately 10 weeks (23 April–30 June 2020).

## METHOD

### Participants and design

The Ethics Review Board of the University of Amsterdam approved this study (2020‐SP‐12194). Participants were recruited via a research panel (Ipsos). The sample of the first wave was representative of the Dutch population in terms of age, gender and residential area. Table 1 provides sample and descriptive information for the longitudinal sample in the column on Wave 5 (valid *N* = 2399), consisting of respondents that participated throughout the study and finished all surveys. This sample size far exceeds the advised sample size of approximately 500 participants for accurate network estimation of a moderately sized network, that is, an accurate representation of the true underlying network (Epskamp, 2017; van Borkulo et al., 2014).

**TABLE 1:** Methodological and sample information for each separate wave

	Measure	Wave 1	Wave 2	Wave 3	Wave 4	Wave 5
Sample formation						
Start data collection (2020)		23rd April	13th May	27th May	10th June	24th June
End data collection (2020)		5th May	18th May	2nd June	16th June	30th June
Failed attention check^a^	*n*	519	118	41	24	8
Sample (including missing values)	*n*	6219	4953	3754	2822	2449
Dropout^b^	*n* (%)		1266 (20.4%)	1199 (24.2%)	932 (24.8%)	373 (11.4%)
Missing values^c^	*n* (%)	126 (2.0%)	89 (1.0%)	70 (1.9%)	58 (2.1%)	50 (2.0%)
Valid *N*		6093	4864	3684	2764	2399
Demographics valid *N*						
Gender	% female	51.4%	50.6%	50.1%	49.1%	49.4%
Age	Range (years)	16–89	16–89	18–89	18–89	18–89
	*M (SD*)	49.32 (16.72)	51.20 (16.32)	51.99 (16.30)	53.42 (16.06)	53.69 (15.84)
Education	% primary or secondary education	55.3%				54.6%
	% higher education	44.7%				45.4%
Illness	% confirmed	30.6%				34.9%
Smoking	% confirmed	17%				15.9%

^a^ Two attention checks were included in each wave to ensure data quality. Failing both attention checks led to exclusion from further data collection and analysis in all subsequent waves.

^b^ Comparing networks of the longitudinal sample and dropouts showed very few significant differences in relations between variables. More information is provided in the Supporting Information (S1.1).

^c^ The answer option ‘I prefer not to answer’ was included in items on education, illness and smoking, and was treated as a missing value. Participants with missing values for one or more nodes were deleted from network analysis, which is reasonable given the small number of missing values.

### Measures

Data collection was conducted with a Dutch online survey. We first identified the most important constructs in the literature (see introduction) and then developed a survey with items based on these constructs (see Supporting Information S1.2 for a complete overview). Analysis for node construction was conducted with the largest and most diverse sample available, namely wave 1, as this wave included all participants that completed the first survey (i.e. featured no dropout). Psychological variables were constructed as nodes through either predetermined combinations of items or based on results of a reduction technique aimed at identifying components in data (i.e. principal axis factoring [PAF]). Detailed descriptions of how items were combined to form nodes, including PAF results, are provided in Supporting Information S1.3. Resulting nodes are presented in Table 2.

**TABLE 2:** Nodes (psychological variables) based on items in the survey, including item examples and answer scales

Node (items per node)	Examples of items per node (/ in the same text line means separate item in survey)	Scale
Compliance (5)	I comply with the corona measures./Keep 1.5 metres away from others./Wash your hands regularly with water and soap	1 (*I do not display this behaviour more*) to 7 (*I display this behaviour much more now*)
Risk Perception (2)	How likely (/severe) do you believe it is you will get infected with the coronavirus within the next year?	1 (*Extremely unlikely*) to 7 (*Extremely likely*)
Health Risk (2)	For me personally (/my family and friends), I consider the health risk of an infection with the coronavirus…	1 (*Extremely small*) to 7 (*Extremely severe*)
Economic Consequences (2)	For me personally (/my family and friends), I consider the economic consequences of the corona pandemic…	1 (*Extremely small*) to 7 (*Extremely severe*)
Self‐exempting Beliefs (2)	I think I am already immune (protected) against the coronavirus./I will not get infected with the coronavirus because I never get the seasonal flu either	1 (*Strongly disagree*) to 7 (*Strongly agree*)
Negative Affect (8)	The corona pandemic is making me (feel)… (e.g. sad/frustrated/overwhelmed)	1 (*Strongly disagree*) to 7 (*Strongly agree*)
Compassion (1)	The corona pandemic is making me feel compassion	1 (*Strongly disagree*) to 7 (*Strongly agree*)
Worries Virus^a^ (4)	I worry about.. (e.g. getting infected/infecting others/the health care system overloading)	1 (*Do not worry at all*) to 7 (*Worry a lot*)
Worries Measures^a^ (6)	I worry about.. (e.g. a recession/limited access to food/getting lonely)	1 (*Do not worry at all*) to 7 (*Worry a lot*)
Vaccination Intention^a^ (1)	If a vaccine becomes available, I would get it	1 (*Strongly disagree*) to 7 (*Strongly agree*)
Measures Support (7)	I find the corona measures.. (Unnecessary–Necessary/Unfair–Fair/Unacceptable–Acceptable)	1 (Negative option) to 7 (Positive option)
Measures Ease (2)	I find the corona measures.. (Difficult–Easy/Unpleasant–Pleasant)	1 (Negative option) to 7 (Positive option)
Social Norm (2)	I think the majority of people (/find it important that people) comply with the corona measures	1 (*Strongly disagree*) to 7 (*Strongly agree*)
Control Infection (2)	For me personally (/my family and friends), avoiding an infection with the coronavirus in the current situation is..	1 (*Extremely difficult*) to 7 (*Extremely easy*)
Self‐efficacy (1)	I know how to protect myself from the coronavirus	1 (*Strongly disagree*) to 7 (*Strongly agree*)
Involvement (3)	How important is the topic of corona pandemic to you?/To what extent does the news about the corona pandemic have your attention?	1 (*Not at all*) to 7 (*Very much*)
Perceived Knowledge (1)	How much knowledge do you think you have about the corona pandemic?	1 (*Very little*) to 7 (*Very much*)
Trust (4)	I trust.. (e.g. the authorities/health care professionals).. during the corona pandemic	1 (*Strongly disagree*) to 7 (*Strongly agree*)
General Health (1)	In general, how would you rate your health?	1 (*Very poor*) to 7 (*Very good*)
Health change Physical (1)	How would you rate your physical health now as compared to before the corona pandemic?	−3 (*Much worse*) to 3 (*Much better*)
Health change Mental (1)	How would you rate your mental health now as compared to before the corona pandemic?	−3 (*Much worse*) to 3 (*Much better*)
Healthy Lifestyle (3)	I've been (exercising/eating/sleeping) in the past two weeks, compared to before the corona pandemic…	−3 (*Much [less healthy/less/worse]*) to 3 (*Much [healthier/more/better]*)
Mental Well‐being^b^ (7)	I've been feeling useful./I've been dealing with problems well./I've been feeling close to other people	1 (*Never*) to 5 (*Always*)
Loneliness^c^ (6)	There are plenty of people I can lean on when I have problems./There are enough people I feel close to	1 (*Not at all*) to 5 (*Very much*)
Complaints^d^ (anxiety [6]; depressive [6]; somatic [6])	To what extent did you experience.. (e.g. feeling suddenly scared/fearful/tense; e.g. feeling lonely/hopeless/a loss of interest; e.g. difficulty breathing/numbness/feeling weak) during the past two weeks?	1 (*Not at all*) to 5 (*Very much*)

*Note*: Mean scores of items were calculated for nodes that were based on multiple items (except for risk perception as explained in text). The sections of the survey that referred to ‘the corona measures’ contained the following explanatory text ‘By this, we mean the recommendations to prevent the spread of the coronavirus and thus prevent overloading the healthcare system, for example stay at home as much as possible, keep 1.5 metres of distance from others and wash your hands regularly with soap and water’.

^a^ WHO Regional Office for Europe (2020), Multiple items (e.g. worries, vaccination intention) were adopted from the WHO protocol for COVID‐19 monitoring.

^b^ Tennant et al. (2007), raw scores were converted to metric scores as required for the (S)WEMWBS.

^c^ de Jong Gierveld and van Tilburg (2008), answer scale formally ranges from 1 (*No!*) to 5 (*Yes!*). Please note that answer scales were adjusted, and a 2‐week time frame was specified for the current study.

^d^ Derogatis (2001), answer scale formally ranges from 0 (*Not at all*) to 4 (*Extremely*). Please note that we changed the BSI‐18 answer scales and specified a 2‐week time frame, possibly invalidating clinical interpretation of scores. Therefore, we use the term *complaints instead of symptoms*.

#### Compliance

Protective behaviours as recommended by the Dutch national government (Government of the Netherlands, 2020) provided the operationalization of compliance with behavioural measures in this study. We measured to what degree participants adopted protective behaviours as advised to the general public (i.e. physical distancing and hygiene behaviours), providing a self‐reported measure for compliance. Items forming this node changed during the study because of changes in recommended protective behaviours.^1^

#### Attitudes

Attitudes were measured in line with the multi‐component model, namely consisting of cognitive, affective and behavioural attitude elements (Eagly & Chaiken, 1993). Items forming the cognitive attitudinal nodes Risk Perception, Health Risk (*r*_sb_ = .59)^2^ and Economic Consequences (*r*_sb_ = .75) were based on prior research into psychological COVID‐19 networks (Chambon, Dalege, Elberse, et al., 2022). Risk perception was measured with two items on perceptions of likelihood and severity of getting infected with the coronavirus. Risk Perception was the product of these two items (Wolff et al., 2019). Health Risk represents perceived health consequences due to an infection and the node Economic Consequences contains items measuring expected consequences of the pandemic for the economy. Self‐exempting Beliefs (*r*_sb_ = .57) tapped into one's conviction of not being susceptible to an infection with the coronavirus.

Items measuring affect surrounding the pandemic resulted in the nodes Negative Affect (e.g. anxiety, anger and confusion; *a* = .89) and Compassion (single item). Items on worries also resulted in two nodes: Worries Virus (*a* = .73) and Worries Measures (*a* = .67), reflecting worries about events during the pandemic resulting directly from the coronavirus (e.g. losing someone they love) and events resulting from measures taken because of the virus (e.g. a recession), respectively.

Behavioural attitudinal nodes consisted of Vaccination Intention (single item), Measures Support (*a* = .90) and Measures Ease (*r*_sb_ = .68). Vaccination Intention represents one's intention to get a COVID‐19 vaccine once available. The items covering attitudes towards the behavioural measures, consisting of both general items and semantic differential scale items, resulted in two nodes: Measures Support reflects support for the behavioural measures advised to prevent the spread of the coronavirus, and Measures Ease represents participants' perceived ease of complying with these measures.

#### Additional psychological factors

Social Norm (*r*_sb_ = .76) reflects prescriptive (what other people think one should do) and descriptive (what other people do) social norms regarding compliance with behavioural measures. Items on perceived control formed the nodes Control Infection (*r*_sb_ = .61) and Self‐efficacy (single item). The former node represents to what degree participants felt able to avoid getting infected with the coronavirus. The latter node represents whether participants perceived to know how to protect themselves from the coronavirus. Involvement (*a* = .84) was comprised of items on how actively involved participants perceived themselves to be in the COVID‐19 pandemic (e.g. watching the news). This node also represents a dimension of attitude strength. A single item measuring perceived knowledge about the pandemic formed the node Perceived Knowledge. The node Trust (*a* = .86) reflects general trust in the four actors relevant to the corona pandemic in the Netherlands: the authorities, the Dutch National Institute for Public Health and the Environment (RIVM), health care professionals and science.

#### Physical and mental health nodes

General Health reflects an overall self‐reported evaluation of participants' health. Single items measuring participants' evaluation of their physical and mental health during the survey, compared to before the pandemic, formed the nodes Health change Physical and Health change Mental, respectively. Lifestyle items measured an improvement or deterioration in exercise, diet and sleep, resulting in Healthy Lifestyle (*a* = .49).^3^ The sum of items from the Short Warwick Edinburgh Mental Well‐being Scale ([S]WEMWBS; Tennant et al., 2007) formed Mental Well‐being (*a* = .84). The shortened version of the loneliness scale (de Jong Gierveld & van Tilburg, 2008) resulted in the node Loneliness (*a* = .76). The Brief Symptom Inventory 18 (BSI‐18; Derogatis, 2001) was adopted to measure psychological complaints. This scale contains 18 items measuring three areas of psychological distress (i.e. anxiety, depressive and somatic symptoms). Three nodes were formed based on the BSI‐18 items: Anxiety Complaints (*a* = .92), Depressive Complaints (*a* = .90) and Somatic Complaints (*a* = .84).

#### Individual differences

The first survey also included questions on individual differences, namely demographics (i.e. age/gender/education) and health (i.e. illness/smoking). These variables provided descriptive information about the sample and were excluded from temporal analyses given that they were measured just once, in the first wave. The first survey also included items on relatively stable personality aspects (i.e. consideration of future consequences, resilience and coping) that provided input for a different paper on interventions. Readers are referred to Chambon, Dalege, Waldorp, et al. (2022) for more information on (results of) these measures.

### Procedure

Participants that subscribed to Ipsos' research panel received an invitation to participate via e‐mail. Participants were informed about and asked to commit to the longitudinal research design beforehand. Only participants who finished the survey received invitations for subsequent waves. They received compensation in the form of points that can be spent at web shops.

Interventions were also included at the beginning of the third and last wave, to which participants were randomly assigned (see Supporting Information S1.4). These interventions are not the focus of the current paper and will be presented in a different paper Chambon, Dalege, Waldorp, et al. (2022).

### Network analysis

Networks were estimated with the *dlvm1* function (Lag‐1 dynamic latent variable model for panel data) in *psychonetrics* (Epskamp, 2020a, 2020b). The R script is made available in the Supporting Information (S1.5). We estimated two networks that provide information on average within‐person effects on a population level: *temporal* and *contemporaneous* (Epskamp, 2020a) psychological networks.^4^ Temporal networks contain edges representing whether one node predicts other nodes in the next measurement while controlling for all other nodes. These temporal effects are calculated by regressing each variable on all variables (including itself) on the previous measurement (i.e. lag‐1) and therefore require repeated measures (i.e. waves). The resulting partial correlations are indicative of directed predictive effects, depicted by edges with arrows in the network (from wave t‐1 to wave t). The weight of edges indicates their effect size and can be interpreted as one would interpret regular partial correlations. Edges between two nodes represent either a causal effect or are the result of a third (unknown) underlying cause. Contemporaneous networks are based on residuals of the temporal network (i.e. variance and covariance that cannot be explained by the modelled temporal effects). These edges can be interpreted as partial correlations between nodes in the same measurement after controlling for all other nodes in the same measurement and the previous measurement (i.e. temporal effects). These partial correlations thus represent undirected associations, depicted by edges without arrows in the network. Again, edge weights indicate effect size and can be interpreted as regular partial correlations. Readers are referred to Epskamp et al. (2018) for additional information on the type of networks presented here.

Centrality measures facilitate the interpretation of visualized networks. The centrality measure ‘strength’ is among the most commonly used centrality measure for psychological networks (Borsboom et al., 2021) and provides information on the conditional association between a node and other nodes in the network. It is calculated by the sum of the absolute edge weights of relations a specific node has with connected nodes. Two different types of strength can be distinguished in temporal networks, due to directed edges: InStrength for edges directed towards that specific node and OutStrength for edges directed from that specific node to other nodes. In other words, we distinguish between effects *to* a node and *from* a node.

## RESULTS

This section presents COVID‐19 broad attitude networks containing temporal dynamics based on the longitudinal survey. In the current study, temporal effects indicate which nodes predict other nodes over a time frame of 2–3 weeks (i.e. from one survey of a wave to the next), whereas contemporaneous effects indicate which nodes predict other nodes within the same survey of a wave.

### Preliminary analysis

**FIGURE 2:**
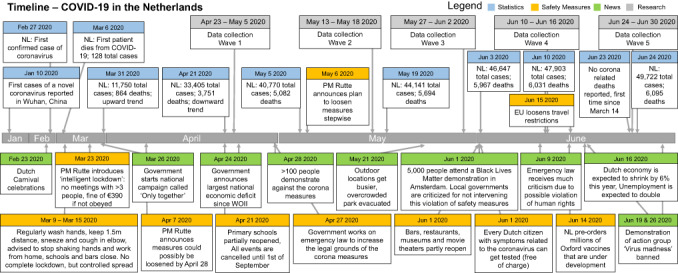
Timeline of important events in the Netherlands during data collection. PM = Prime Minister. Contains relevant statistics (blue), safety measures (orange) and news items (green) related to the COVID‐19 pandemic, including the moments of data collection for each wave (grey). Please note that a downward trend of the number of COVID‐19 infections started before the data collection started

Descriptive statistics of nodes are provided in Supporting Information S2.1, together with results of repeated measures ANOVA analyses to examine the effects of time on variables. Results indicated a significant change in all nodes over time. Furthermore, Figure 2 provides the context at the time of data collection by showing a general timeline of the pandemic in the Netherlands (see Supporting Information S3 for underlying data).

Evaluation of the overall fit of the *dlvm1* model in which we included all edges in the COVID‐19 broad attitude networks showed excellent fit (see Supporting Information S2.2). Generally speaking, confidence intervals of edge weights were not wide, indicating stable (reliable) edge estimates. The weights of edges discussed below are reported in parentheses (see Supporting Information S2.3–S2.5 for a complete overview of edges in the COVID‐19 networks).

### Temporal COVID‐19 network

**FIGURE 3:**
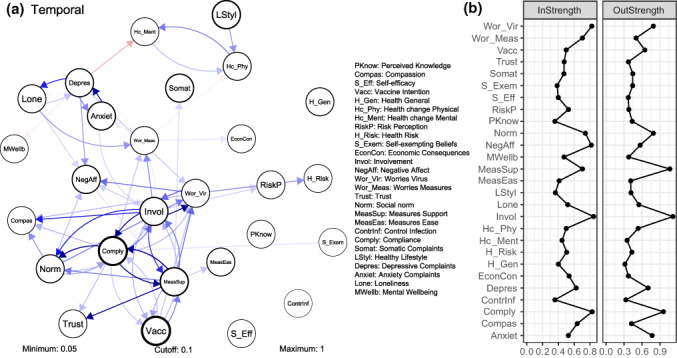
The temporal COVID‐19 network. (a) Visualized network—See Table 3 for edges weights. Nodes represent measured psychological factors. Edges represent relations between two nodes after controlling for other nodes in the network, with their weight indicating the strength of relations. Blue edges represent positive (excitatory) relations and red edges represent negative (inhibitory) relations. Edges with weights below .05 are omitted to facilitate readability. The network has a cut‐off value of .10, meaning edges with weights below that value are depicted with similar width and colour density; (b) standardized strength measure. This measure represents direct effects of a specific node on the network and is calculated by the sum of the absolute edge weights, with InStrength for edges affecting that node and OutStrength for edges from that node affecting other nodes

Figure 3a shows temporal effects, standardized to partial directed correlations, in the COVID‐19 broad attitude network. Edges indicate nodes' predictive value for the next measurement after controlling for all other nodes. Table 3 provides edge weights of all edges in the temporal network. The width of node borders represents the degree to which nodes were influenced by the same node in the previous wave (i.e. autoregression). Thicker node borders indicate more stable nodes. The most stable nodes in the temporal COVID‐19 network were Vaccination Intention (.41), Compliance (.37), Measures Support (.25) and Healthy Lifestyle (.24).

**TABLE 3:**
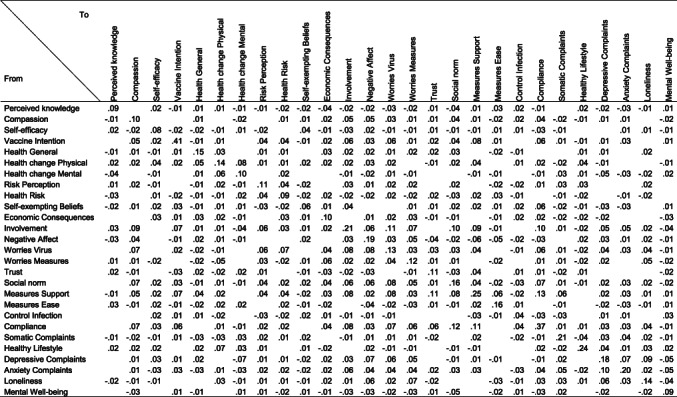
Edge weights of partial directed correlations from the temporal COVID‐19 network

*Note*: Read rows (first column) as node from which the edge originates.

Regarding nodes related to compliance, results showed bidirectional effects between Compliance and multiple other variables in the network after controlling for the effects of every other node in the network. Compliance was predicted by Measures Support (.13), Involvement (.10), Social Norm (.07), Vaccination Intention (.06) and Worries Virus (.06). Also, Compliance predicted Social Norm (.12), Measures Support (.11), Involvement (.08), Worries Virus (.07) and Vaccination Intention (.06). The former indicates that change in compliance with behavioural measures was predicted by the degree to which one supports the behavioural measures, was mentally involved in the COVID‐19 pandemic, perceived social norms regarding compliance, intended to get vaccinated against COVID‐19 and worried because of the virus. The latter indicates that change in compliance with behavioural measures was predictive of one's perceived social norms regarding compliance, support for the behavioural measures, mental involvement in the pandemic, worries because of the virus and intention to get vaccinated against COVID‐19. Such positive feedback loops indicate that compliance decreases over time if one of the aforementioned five variables decreases, and, vice versa, that these five variables decrease if compliance decreases. Figure 3a shows that the temporal network contains more of these patterns.

Moreover, results suggest bidirectional effects between three of the variables that showed bidirectional effects with compliance. That is, the nodes Measures Support, Involvement and Vaccination Intention had bidirectional relations among each other. Such a pattern of relations between Compliance, Measures Support, Involvement and Vaccination Intention suggests a reinforcing structure between these nodes.

Another interesting pattern in the temporal network was found between Depressive Complaints, Anxiety Complaints and Loneliness. Depressive Complaints predicted Anxiety Complaints (.07) and Loneliness (.09), and Anxiety Complaints and Loneliness predicted Depressive Complaints (.10 and .06, respectively). However, edges between Anxiety Complaints and Loneliness were relatively small (i.e. from Anxiety Complaints to Loneliness .02; from Loneliness to Anxiety Complaints .03). This pattern suggests a relatively central role for Depressive Complaints in this triangle with Anxiety Complaints and Loneliness.

#### Node centrality

Figure 3b shows the standardized centrality measure ‘strength’ for the temporal COVID‐19 network (value is provided in text in parentheses). Results showed that Involvement, Compliance, Worries Virus and Social Norm had a central role in the COVID‐19 network by both affecting and being affected by other nodes (OutStrength 2.48, 1.79, 1.05, 1.05; InStrength 1.87, 1.75, 1.72, 1.21, respectively). Interestingly, Measures Support predominantly affected other nodes (OutStrength 2.26) and was affected to a lesser extent (InStrength 0.97). Salient discrepancies between InStrength and OutStrength measures were found for Negative Affect, which was predominantly affected by other nodes in the COVID‐19 network (InStrength 1.69; OutStrength 0.08) and Worries Measures, which was mostly affected by other nodes (InStrength 0.97; OutStrength −0.22). Finally, nodes relevant to mental health were of relatively low strength, suggesting that these nodes had a limited impact on the network. A table with node strength values for each node is provided in Supporting Information S2.4.

### Contemporaneous COVID‐19 network

**FIGURE 4:**
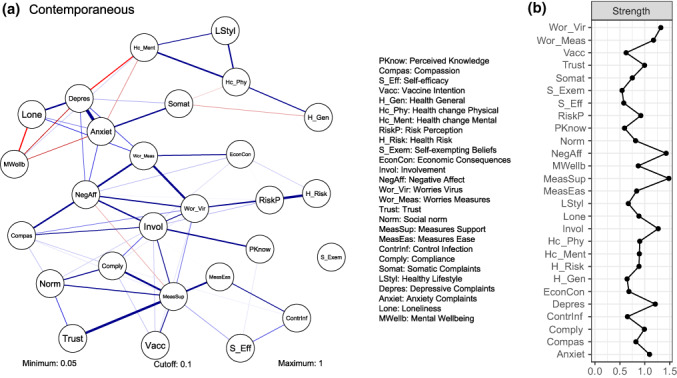
The contemporaneous COVID‐19 network. See Figure 3 for interpretation guidelines. (a) Visualized network. Positive (negative) edges indicate that people who reported higher scores on one variable also reported higher (lower) scores on the other variable. See S2.4 for the corresponding edge weights table; (b) standardized centrality measures

Figure 4 shows (a) contemporaneous effects in the COVID‐19 broad attitude network (left) and (b) the standardized centrality measure ‘strength’ for the contemporaneous COVID‐19 network (right). Results concerning edges related to Compliance are comparable to the temporal level: the strongest edges with Compliance are Measures Support (.22), Social Norm (.13) and Involvement (.09). These edges indicate that when someone reports compliance, they are also likely to report support for the measures, social norms and involvement in the pandemic. This, together with the effects in the temporal network (see Figure 3a), implies that dynamics concerning compliance with behavioural measures are comparable between measurements over time (i.e. temporal) and within measurements (i.e. contemporaneous). Strength measures for the node Compliance differed between the COVID‐19 broad attitude networks: this node played a more central role in the temporal network (InStrength 1.75; OutStrength 1.79) than in the contemporaneous network (0.32). This indicates that, relative to other nodes, compliance with behavioural measures is more predictive of and predicted by other nodes in the network in the next wave than that it was strongly connected with other nodes in the same survey.

Furthermore, strength measures of the contemporaneous COVID‐19 network showed that several variables with higher strength in the temporal network also played a central role here, namely Measures Support (2.16), Negative Affect (1.96), Worries Virus (1.56) and Involvement (1.36). This implies that these nodes are important not only for the COVID‐19 broad attitude network over time between measurements (i.e. the nodes are predictive of and/or predicted by many other nodes in the network) but also within a measurement wave (i.e. these nodes are related to many other nodes in the same survey). Interestingly, the node Social Norm had relatively high strength on the temporal level (InStrength 1.21; OutStrength 1.05) but less on the contemporaneous level (−0.35). This suggests that social norms are more predictive of other nodes in the COVID‐19 broad attitude network over time than that this node was strongly connected with other nodes in the same survey.

Finally, we conducted correlational analyses to examine coherence between the temporal and contemporaneous networks. Results showed a moderate to large correlation between the temporal and contemporaneous COVID‐19 networks (*r* = .47, *z* = 0.51; see Supporting Information S2.6 for plot). This indicates a relation between edges representing effects over time between measurements (i.e. waves) and edges representing effects during measurement (i.e. within a survey).

## DISCUSSION

The current research explored the temporal dynamics of psychological factors related to compliance with behavioural measures during the initial phase of the COVID‐19 pandemic. Results of this high‐powered longitudinal study showed that nodes in the broad attitude networks were highly interconnected. This indicates that all included variables provided substantive information on the dynamics of adopting protective behaviours during a pandemic, justifying a complexity approach. Several insights can be drawn from this study.

Three interesting patterns were observed in the temporal network. First, results show a series of bidirectional effects between variables in the temporal network relevant for compliance with behavioural measures during the COVID‐19 pandemic. We found that the degree to which people support behavioural measures and were mentally involved in the pandemic were most important for predicting compliance over time after controlling for every other variable in the network. This is in line with previous research showing relations between support for measures and compliance (Chambon, Dalege, Elberse, et al., 2022; van Rooij et al., 2020). Interestingly, the current study also found effects the other way around, namely that compliance predicted support for the behavioural measures and involvement in the pandemic over time. Similar results were obtained for vaccination intention and social norms: not only do these variables predict compliance with behavioural measures over time, but compliance also predicts these variables over time. These potential feedback loops between variables consist of positive relations between two nodes, indicating that effects strengthen as time progresses. More specifically, an increase in one variable leads to an increase in the other variable in the next measurement, which in turn leads to an increase in the first variable in the measurement thereafter, etcetera. Insights regarding such potential feedback loops add to existing literature such as the conceptual frameworks of health behaviour during a pandemic proposed by Bish and Michie (2010) and underlying theoretical frameworks such as the TPB, HBM and PMT by moving from unidirectional effects to more complex interactions between variables. Moreover, such feedback loops provide possible explanations for observed effects. For instance, we observed a decrease in compliance with behavioural measures, support for these measures and involvement in the pandemic throughout the course of this study. According to the temporal network, a decrease in compliance could be explained by a decrease in support for the measures or involvement. The feedback loops suggest that this applies vice versa as well: a decrease in support for the measures or involvement could be explained by a decrease in compliance. These findings resonate with different theoretical models in which behaviour can both be determined by other variables (e.g. TPB; Ajzen, 1991) and determine other variables (e.g. dissonance theory; Festinger, 1957). Moreover, the findings suggest that (elements of) these models can apply simultaneously. Thus, combining different models with bidirectional effects between variables might improve our understanding of health behaviour in a pandemic beyond unidirectional effects between limited sets of variables based on singular theoretical models.

The second pattern observed in the temporal network is that several of these bidirectional effects combined formed a positive reinforcing structure. That is, three variables that showed positive feedback loops with compliance (i.e. support for behavioural measures, involvement in the pandemic and vaccination intention) also showed positive feedback loops among each other. Such structures can potentially amplify change in these variables. That is, an increase (or decrease) in one variable from the reinforcing structure is likely to be accompanied by an increase (or decrease) in the remaining variables over time, further increasing (decreasing) the initially increased (decreased) variable and other variables over time, and so on.

Such positive reinforcing structures are important to acknowledge for different scenarios. First, considering interventions: if one aims to increase compliance, doing so via a variable that is not only directly related to compliance but also part of a positive reinforcing structure could amplify the effects of interventions over time. For instance, results suggest that an intervention aimed at positively influencing support for behavioural measures could increase not only compliance but also involvement in the next time frame. This could increase compliance via multiple variables: in addition to the intervention initiating a feedback loop between compliance and support for the measures, the intervention's effect on involvement could also initiate the feedback loop between compliance and involvement. Thus, positive reinforcing structures can amplify the effects of interventions beyond bidirectional effects.

The second reason why reinforcement structures are important is that they can provide insight into processes of polarization during a pandemic. Reinforcement structures can enhance initial leanings towards one end of the evaluative spectrum and lead to a movement towards the extremes of that spectrum. This effect resembles the phenomena of polarization, in which people strengthen their attitude in the initial direction, a process that has been reported in relation to the initial phase of the COVID‐19 pandemic (Graso et al., 2021; Kerr et al., 2021). The observation that involvement is part of a reinforcing structure with compliance, support for behavioural measures and vaccination intention corresponds with the Attitudinal Entropy (AE) framework grounded in network theory (Dalege et al., 2018). This framework explains polarization through attitudinal entropy, a state reflecting inconsistent and instable attitudes. Dalege et al. (2018) propose that attitudinal entropy is reduced by thinking about and turning attention towards attitudes objects, and involvement as included in the current study can be interpreted as such. This reduction in entropy results in strengthening attitudes in the initial direction, i.e. polarization. This is also in line with the mere thought effect on attitude polarization (Tesser & Conlee, 1975), proposing that thought can result in attitude polarization.

The third pattern observed in the temporal network is also relevant in the context of interventions. That is, the network contained a triangle of three nodes in which one node shows a bidirectional relation with both other nodes, but these other nodes show a weak or no relation with each other. Such a pattern was for instance observed between depressive complaints, anxiety complaints and loneliness, in which depressive complaints showed a bidirectional relation with both other nodes, but anxiety and loneliness showed a relatively weak relation. This suggests that intervening on depressive complaints can be effective to improve mental health.

Another insight from this study is that our results suggest a weak predictive effect between (nodes related to) mental health and compliance. One could have expected a predictive relation, for example by compliance in the form of adhering to social distancing and isolation guidelines to have an impact on mental health, but there were only weak predictive effects between these constructs when controlling for other variables. This could imply that relations between variables related to mental health (e.g. stress) and compliance observed in prior research (Harper et al., 2021; Lieberoth et al., 2021) might be explained by constructs other than included in those studies, for instance, support for the behavioural measures or involvement in the pandemic. Another possible explanation could be that prior research focussed mainly on between‐person effects and that relations between mental health and compliance are more pronounced on that level. Comparing psychological within‐person and between‐person effects in the context of compliance and mental health during pandemics would be the next step in this line of research to examine whether meaningful differences can be observed.

A final insight of this study is that the most important temporal effects concerning compliance with behavioural measures between measurements were comparable with effects found within the same measurement (i.e. contemporaneous effects). For instance, the positive reinforcing structure found between compliance, support for the behavioural measures, involvement and vaccination intention was found not only over time between measurements but also within the same measurement: higher scores on compliance, support for the measures, involvement in the pandemic and vaccination intention tends to co‐occur during a measurement. However, there were also important differences between these time frames, as corroborated by a moderate to large relation between the (edges in the) networks. These differences are also important to consider when designing interventions as these can be tailored based on their anticipated effects. For instance, social norms were found to be relatively important for the COVID‐19 network over time but not within the same measurement. This implies that the effects of interventions aimed at influencing social norms should ideally be studied over time, that is, a next measurement and not within the same survey.

The current research enables us to formulate several recommendations. The first recommendation concerns future research into causality. Although interesting indications of causality can be drawn based on the approach adopted in this study, no firm statements on causality can be made. In order to do so, interventions should be studied (Kossakowski et al., 2021). Second, the ecological validity of the presented COVID‐19 broad attitude networks is unknown. Although the COVID‐19 broad attitude networks contain a diverse set of variables, more psychological variables can be relevant for compliance with behavioural measures during pandemics. Changes over time observed in this study could also be caused by predictors not included in the model. This also applies to the contemporaneous effects, which can also indicate that other variables have not (yet) been taken into account. Future research could include additional variables that are deemed relevant based on the scientific literature to further meet real‐life complex psychological systems concerning pandemics. Third, although comparing networks of the longitudinal sample and other respondents showed very few significant differences in relations between variables, we cannot predict how respondent dropout affects the results presented here. Given the aim to demonstrate how this approach provides an overview of predictive relations between variables, and not to present a generalizable theoretical model or representative overview of node scores, we assume that dropout has no significant effect on the outcome given the aim of the current study. Fourth, future research could examine temporal dynamics of compliance during pandemics in different phases of a pandemic. The current study was conducted during the initial phase of the COVID‐19 pandemic and we cannot predict to what degree results are generalizable beyond the first wave. Results therefore represent a particular period in time, and future research could focus on other phases such as the perseverance period in which adopting protective behaviours needs to be maintained. Fifth and finally, it should be noted that the time spacing of measurements is likely to affect results. For example, it is more likely that change in relatively stable nodes such as compliance and intention to get vaccinated is detected when administering surveys weeks apart than hours apart. We think that the time frame adopted in this study (i.e. 2 to 3 weeks between surveys) is adequate to detect changes in this context; nevertheless, future research could adopt different time frames to examine whether this generates different patterns of effects.

In conclusion, the COVID‐19 broad attitude networks obtained in this study show the added value of adopting a complexity approach to compliance in the context of pandemics. Moreover, the adopted method provides insight into unique relations between a broad set of variables, and how relations between these variables develop over time. Finally, the results suggest that the network structure can provide important insights for explaining observed effects and designing effective interventions, providing an informed strategy grounded in network theory to influence compliance during pandemics.

## AUTHOR CONTRIBUTIONS

**Monique Chambon:** Conceptualization; data curation; formal analysis; investigation; methodology; project administration; validation; visualization; writing – original draft. **Jonas Dalege:** Formal analysis; methodology; validation; writing – review and editing. **Denny Borsboom:** Methodology; validation; writing – review and editing. **Lourens J. Waldorp:** Methodology; validation; writing – review and editing. **Han L.J. Van der Maas:** Methodology; validation; writing – review and editing. **Frenk van Harreveld:** Conceptualization; funding acquisition; investigation; methodology; project administration; supervision; validation; writing – review and editing.

## CONFLICT OF INTEREST

The author(s) declared no potential conflicts of interest.

### OPEN RESEARCH BADGES

This article has earned Open Data and Open Materials badges. Data and materials are available at https://osf.io/qu7p2/.

## Supporting information


Appendix S1

## Data Availability

The data that support the findings of this study is made openly available in OSF at https://osf.io/qu7p2/. R code is also made available on OSF. Supplemental materials are available in the online version of the article.

## Footnotes

^1^
The node Compliance consisted of five items in the first wave (*a* = .75), and one of these items was adapted for the second wave. A sixth item was added in the third wave, leading to Compliance consisting of six items from the third wave onwards. Thus, this node contains behavioural items that were applied during the administration of the survey.

^2^
The scale reliability, although for some nodes lower than one would normally prefer, was interpreted as sufficient since our aim was to measure evaluations and not to design reliable measuring scales (Dalege et al., 2016).

^3^
See previous footnote.

^4^
The analysis normally also provides a between‐person network, but estimating this network was not successful as the edges in this network had too high standard errors to be interpretable. Confidence intervals that are too wide can result in unstable (unreliable) edges; thus, this network was omitted in the current paper.
