# Supplementary material for: How compliance with behavioural measures during the initial phase of a pandemic develops over time: A longitudinal COVID‐19 study
Source: Br J Soc Psychol. 2022 Oct 10;62(1):302–21. doi: 10.1111/bjso.12572 (PMC9874881; doi:10.1111/bjso.12572)
Supplement: Supplementary file 1 — Appendix S1 [file BJSO-62--s001.docx]

Supplement COVID-19 study

*How compliance with behavioural measures during the initial phase of a pandemic develops over time: a longitudinal COVID-19 study*

Contents

[1. Method 2](#_Toc106710709)

[1.1 Dropped out participants longitudinal study 2](#_Toc106710710)

[1.2 Survey items per node in the COVID-19 broad attitude networks 3](#_Toc106710711)

[1.3 Node construction and Principal Axis Factoring 7](#_Toc106710712)

[1.4 Comparing results from intervention conditions 12](#_Toc106710713)

[1.5 R script 14](#_Toc106710714)

[2. Results 15](#_Toc106710715)

[2.1 Descriptive statistics and Repeated Measures ANOVA 15](#_Toc106710716)

[2.2 Network analysis model fit evaluation 23](#_Toc106710717)

[2.3 COVID-19 broad attitude networks including all edges 25](#_Toc106710718)

[2.4 Edge weights and node strength of COVID-19 broad attitude networks 26](#_Toc106710719)

[2.5 Edge accuracy of COVID-19 broad attitude networks 30](#_Toc106710720)

[2.6 Coherence within-person COVID-19 broad attitude networks 32](#_Toc106710721)

[3. Timeline COVID-19 in the Netherlands 33](#_Toc106710722)

[4. References 48](#_Toc106710723)

# Method

## 1.1 Dropped out participants longitudinal study

Demographic information was compared between the longitudinal sample and the participants that dropped out of the study before the final wave (total drop-out *n* = 3,694). A chi square test of independence indicated that the proportion of participants that dropped out of the study differed by gender, $\chi^{2}$ (1, *N* = 6,093) = 6.55, *p* = .011, and illness, $\chi^{2}$ (1, *N* = 6,093) = 34.53, *p* < .001. Specifically, men and participants suffering from a condition that could worsen the consequences of COVID-19 were more likely to participate in the current study up and until the final wave. No significant differences between the longitudinal sample and drop-outs were found for education and smoking. A Mann-Whitney *U* test indicated that the age was significantly higher in the longitudinal sample (*Mdn* = 56) than in the group of participants that dropped out of the study (*Mdn* = 47), *U* = 5,529,364.50, *p* < .001, which implies that the average age of the longitudinal sample is higher than the average Dutch population.
 The Network Comparison Test (van Borkulo et al., 2022) was conducted to formally test for differences in network structure at the first measurement between respondents from the longitudinal sample and respondents that dropped out of the study after the first measurement. Results revealed network invariance (*p* <.001), indicating that not all edges are equal between the networks of the first measurement of the longitudinal sample and respondents that dropped out. Further investigation into specific edge differences reveals that, after applying a Holm correction for multiple testing, only two edges differ significantly at the first measurement between the longitudinal and dropout sample. More specifically, the edges that differ significantly are somatic symptoms and self-exempting beliefs (i.e., weak positive edge is stronger in dropout sample), and between somatic symptoms and social norm (i.e., weak negative edge is absent in dropout sample). Finally, results indicated a significant difference in global strength (i.e., overall connectivity of the networks) between the longitudinal sample and respondents that dropped out of the study (global strength is 2.61, *p* = .036).

## 1.2 Survey items per node in the COVID-19 broad attitude networks

| Factor | Survey items - (R) = reverse score | Answer scale |
| --- | --- | --- |
| Compliance T1 | I comply with the corona measures. | 1 (*I never comply with the measures*) to 7 (*I always comply with the measures*) |
|  | Stay at home as much as possible. | 1 (*I do not display this behaviour mor*e) to 7 (*I display this behaviour much more now*) |
|  | Keep 1.5 meters away from others. |  |
|  | Wash your hands regularly with soap and water. |  |
|  | Cough and sneeze into the inside of your elbow. |  |
| Compliance T2 | I comply with the corona measures. | 1 (*I never comply with the measures*) to 7 (*I always comply with the measures*) |
|  | Keep 1.5 meters away from others. | 1 (*I do not display this behaviour mor*e) to 7 (*I display this behaviour much more now*) |
|  | Wash your hands regularly with soap and water. |  |
|  | Cough and sneeze into the inside of your elbow. |  |
|  | Avoid crowds. |  |
| Compliance T3 -T5 | I comply with the corona measures. | 1 (*I never comply with the measures*) to 7 (*I always comply with the measures*) |
|  | Keep 1.5 meters away from others. | 1 (*I do not display this behaviour mor*e) to 7 (*I display this behaviour much more now*) |
|  | Wash your hands regularly with soap and water. |  |
|  | Cough and sneeze into the inside of your elbow. |  |
|  | Avoid crowds. |  |
|  | Stay close to home. |  |
| Risk Perception | How likely do you think it is that u will get infected with the coronavirus in the upcoming year? | 1 (*Extremely unlikely*) to 7 (*Extremely likely*) |
|  | How severe do you think an infection with the coronavirus would be for you? | 1 (*Not severe*) to 7 (*Very severe*) |
| Health Risk | For me personally, I consider the health risk of an infection with the coronavirus.. | 1 (*Extremely small*) to 7 (*Extremely severe*) |
|  | For my family and friends, I consider the health risk of an infection with the coronavirus.. |  |
| Economic Consequences | For me personally, I consider the economic consequences of the corona pandemic .. | 1 (*Extremely small*) to 7 (*Extremely severe*) |
|  | For my family and friends, I consider the economic consequences of the corona pandemic are.. |  |
| Self-exempting Beliefs | I won't get infected with the coronavirus because I never get the seasonal flu (influenza) either. | 1 (*Strongly disagree*) to 7 (*Strongly agree*) |
|  | I think I am already immune (protected) against the coronavirus. |  |
| Negative Affect | The corona pandemic is making me angry. | 1 (*Strongly disagree*) to 7 (*Strongly agree*) |
|  | The corona pandemic is making me sad. |  |
|  | The corona pandemic is making me feel confused. |  |
|  | The corona pandemic is making me feel uncertain. |  |
|  | The corona pandemic is making me feel overwhelmed. |  |
|  | The corona pandemic is making me feel frustrated. |  |
|  | The corona pandemic is making me fearful. |  |
|  | The corona pandemic is making me feel out of control. |  |
| Compassion | The corona pandemic is making me feel compassion. | 1 (*Strongly disagree*) to 7 (*Strongly agree*) |
| Worries Virus | I worry about getting infected with the coronavirus. | 1 (*Don't worry at all*) to 7 (*Worry a lot*) |
|  | I worry about infecting others with the coronavirus. |  |
|  | I worry about losing someone I love. |  |
|  | I worry about the health care system overloading. |  |
| Worries Measures | I worry about what staying at home a lot will do to my health. | 1 (*Don't worry at all*) to 7 (*Worry a lot*) |
|  | I worry about the schools closing. |  |
|  | I worry about a recession. |  |
|  | I worry about limited access to food. |  |
|  | I worry about losing my job. |  |
|  | I worry about getting lonely. |  |
| Vaccination Intention | If a vaccine becomes available, I would get it. | 1 (*Strongly disagree*) to 7 (*Strongly agree*) |
| Measures Support | I find the corona measures... | 1 (*Senseless*) to 7 (*Sensible*) |
|  | I find the corona measures... | 1 (*Useless*) to 7 (*Useful*) |
|  | I find the corona measures... | 1 (*Unnecessary*) to 7 (*Necessary*) |
|  | I find the corona measures... | 1 (*Unfair*) to 7 (*Fair*) |
|  | I find the corona measures... | 1 (*Unacceptable*) to 7 (*Acceptable*) |
|  | I think the corona measures will prevent the spread of the coronavirus. | 1 (*Strongly disagree*) to 7 (*Strongly agree*) |
|  | As a society, we must do whatever is necessary to prevent the spread of the coronavirus. |  |
| Measures Ease | I find the corona measures... | 1 (*Unpleasant*) to 7 (*Pleasant*) |
|  | I find the corona measures... | 1 (*Difficult*) to 7 (*Easy*) |
| Social norm | I think the majority of people comply with the corona measures. | 1 (*Strongly disagree*) to 7 (*Strongly agree*) |
|  | I think majority of people find it important that people comply with the corona measures. |  |
| Control Infection | For me personally, avoiding an infection with the coronavirus in the current situation is… | 1 (*Extremely* *difficult*) to 7 (*Extremely* *easy*) |
|  | For my family and friends, avoiding an infection with the coronavirus in the current situation is… |  |
| Self-efficacy | I know how to protect myself from the coronavirus. | 1 (*Strongly disagree*) to 7 (*Strongly agree*) |
| Involvement | How important is the topic of corona pandemic to you? | 1 (*Very unimportant*) to 7 (*Very important*) |
|  | To what extent does the news about the corona pandemic have your attention? | 1 (*Not at all*) to 7 (*Very much)* |
|  | How much do you think about the corona pandemic? | 1 (*Very little*) to 7 (*Very much*) |
| Perceived Knowledge | How much knowledge do you think you have about the corona pandemic? | 1 (*Very little*) to 7 (*Very much*) |
| Trust | I trust the authorities to adequately manage the corona pandemic. | 1 (*Strongly disagree*) to 7 (*Strongly agree*) |
|  | I trust RIVM during the corona pandemic. |  |
|  | I trust health care professionals during the corona pandemic. |  |
|  | I trust science during the corona pandemic. |  |
| Health General | In general, how would you rate your health? | 1 (*Very poor*) to 7 *(Very good)* |
| Health Change Physical | How would you rate your physical health now as compared to before the corona pandemic? | -3 (*Much worse*) to 3 (*Much better*) |
| Health Change Mental | How would you rate your mental health now as compared to before the corona pandemic? | -3 (*Much worse*) to 3 (*Much better*) |
| Healthy Lifestyle | I've been exercising in the past two weeks, compared to before the corona pandemic… | -3 (*Much less*) to 3 (*Much more*) |
|  | I've been eating in the past two weeks, compared to before the corona pandemic... | -3 (*Much less healthy*) to 3 (*Much healthier*) |
|  | I've been sleeping for the past two weeks, compared to before the corona pandemic... | -3 (*Much worse*) to 3 (*Much better*) |
| Mental Wellbeing | I’ve been feeling optimistic about the future. | 1 (*Never*) to 5 (*Always*) |
|  | I’ve been feeling useful. |  |
|  | I’ve been feeling relaxed. |  |
|  | I’ve been dealing with problems well. |  |
|  | I’ve been thinking clearly. |  |
|  | I’ve been feeling close to other people. |  |
|  | I’ve been able to make up my own mind about things. |  |
| Loneliness | I experience a general sense of emptiness. | 1 (*Not at all*) to 5 (*Very much*) |
|  | There are plenty of people I can lean on when I have problems. |  |
|  | There are many people I can trust completely. |  |
|  | I miss having people around me. |  |
|  | There are enough people I feel close to. |  |
|  | I often feel rejected. |  |
| Anxiety Complaints | To what extent did you experience nervousness during the past two weeks? | 1 (*Not at all*) to 5 (*Very much*) |
|  | To what extent did you experience feeling suddenly scared during the past two weeks? |  |
|  | To what extent did you experience feeling fearful during the past two weeks? |  |
|  | To what extent did you experience feeling tense during the past two weeks? |  |
|  | To what extent did you experience anxiety or panic attacks during the past two weeks? |  |
|  | To what extent did you experience restlessness during the past two weeks? |  |
| Depressive Complaints | To what extent did you experience a loss of the will to live during the past two weeks? | 1 (*Not at all*) to 5 (*Very much*) |
|  | To what extent did you experience feeling lonely during the past two weeks? |  |
|  | To what extent did you experience feeling blue during the past two weeks? |  |
|  | To what extent did you experience a loss of interest in things during the past two weeks? |  |
|  | To what extent did you experience feeling hopeless about the future during the past two weeks? |  |
|  | To what extent did you experience a feeling of worthlessness during the past two weeks? |  |
| Somatic Complaints | To what extent did you experience dizziness or faintness during the past two weeks? | 1 (*Not at all*) to 5 (*Very much*) |
|  | To what extent did you experience chest pain during the past two weeks? |  |
|  | To what extent did you experience nausea during the past two weeks? |  |
|  | To what extent did you experience difficulty breathing during the past two weeks? |  |
|  | To what extent did you experience numbness during the past two weeks? |  |
|  | To what extent did you experience feeling weak during the past two weeks? |  |

## 1.3 Node construction and Principal Axis Factoring

Analysis for node construction was conducted with the largest and most diverse sample available, namely wave 1, as this wave included all participants that completed the first survey (i.e., featured no drop-out). Survey items were combined to form nodes: the combination of items was either predetermined by validated scales or a fixed operationalization, or identified through Principal Axis Factoring (PAF; see Table 1). PAF was conducted with Oblimin rotation given the expected intercorrelation between items. Extraction of components was based on eigenvalues greater than one. The results are discussed below. Items presented in italic were excluded from the node.

Table 1 - Overview of nodes and the approach to the selection of items per node

| Nodes | Approach to combining items in node |
| --- | --- |
|  |  |
| Compliance (T1) | Predetermined - Operationalized with the recommended behavioural measures,  PAF confirmed single component |
| Risk Perception | Predetermined by product of likelihood and severity |
| Health Risk | Predetermined – Two item construct |
| Economic Consequences | Predetermined – Two item construct |
| Self-exempting Beliefs | PAF – Single component |
| Negative Affect | PAF – Component identified in items on affect |
| Compassion | PAF – Component identified in items on affect |
| Worries Virus | PAF – Component identified in items on worries |
| Worries Measures | PAF – Component identified in items on worries |
| Vaccination Intention | Predetermined single item node |
| Measures Support | PAF – Component identified in items on attitudes toward corona measures |
| Measures Ease | PAF – Component identified in items on attitudes toward corona measures |
| Social Norm | Predetermined – Two item construct |
| Control Infection | Predetermined – Two item construct |
| Self-efficacy | Predetermined single item node |
| Involvement | PAF – Single component |
| Perceived Knowledge | Predetermined single item node |
| Trust | PAF – Single component |
| Health General | Predetermined single item node |
| Health change Physical | Predetermined single item node |
| Health change Mental | Predetermined single item node |
| Healthy Lifestyle | Predetermined - Operationalized with basic items on diet, exercise and sleep,  PAF confirmed single component |
| Mental Wellbeing | Predetermined - Validated scale |
| Loneliness | Predetermined - Validated scale |
| Somatic Complaints | Predetermined - Validated scale |
| Depressive Complaints | Predetermined - Validated scale |
| Anxiety Complaints | Predetermined - Validated scale |

*Compliance*

Compliance was predetermined through operationalization with the recommended behavioural measures. Kaiser-Meyer-Olkin Measure of Sampling Adequacy was .78 and Bartlett’s test of sphericity was significant, χ²(df = 10) = 8,429.90, *p* < .001, supporting a rationale for performing PAF with the five items for *Compliance* in the first wave (i.e. a general item and four specific behaviours). The results suggested a single component for the items on compliance, resulting in the node *Compliance* with the four items shown below.

| No. | Survey items | Compliance Component load |
| --- | --- | --- |
| 1 | I comply with the corona measures. | .837 |
| 2 | Stay at home as much as possible. | .613 |
| 3 | Keep 1.5 meters away from others. | .735 |
| 4 | Wash your hands regularly with soap and water. | .545 |
| 5 | Cough and sneeze into the inside of your elbow. | .444 |

*Self-exempting Beliefs*

Kaiser-Meyer-Olkin Measure of Sampling Adequacy was .54 and Bartlett’s test of sphericity was significant, χ²(df = 3) = 1,320.13, *p* < .001, not convincingly supporting a rationale for performing PAF with the three items for *Self-exempting Beliefs*. Nevertheless, the results suggested excluding the item ‘I think infections are good for building up group immunity (protection)’, because of its low component load. This is supported by an increase in scale reliability after deletion (three items *a =* .47, if item deleted *a =* .57; resulting in *r_sb_* = .57). As a result, the node *Self-exempting Beliefs* consisted of item 1 and 2 (see below).

| No. | Survey items | Self-exempting Beliefs Component load |
| --- | --- | --- |
| 1 | I won't get infected with the coronavirus because I never get the seasonal flu (influenza) either. | .505 |
| 2 | I think I am already immune (protected) against the coronavirus. | .780 |
| *3* | *I think infections are good for building up herd immunity (protection).* | *.242* |

*Negative Affect and Compassion*

Kaiser-Meyer-Olkin Measure of Sampling Adequacy was .90 and Bartlett’s test of sphericity was significant, χ²(df = 36) = 24,219.84, *p* < .001, supporting a rationale for performing PAF with the nine items covering affect. Compassion was identified as a separate component. This resulted in two nodes: *Negative Affect,* consisting of the items 1 to 8 shown below, and the single-item (no. 9) node *Compassion.*

| No. | Survey items | Negative Affect Component load | Compassion Component load |
| --- | --- | --- | --- |
| 1 | The corona pandemic is making me angry. | .729 |  |
| 2 | The corona pandemic is making me sad. | .475 |  |
| 3 | The corona pandemic is making me feel confused. | .678 |  |
| 4 | The corona pandemic is making me feel uncertain. | .649 |  |
| 5 | The corona pandemic is making me feel overwhelmed. | .472 |  |
| 6 | The corona pandemic is making me feel frustrated. | .890 |  |
| 7 | The corona pandemic is making me fearful. | .572 |  |
| 8 | The corona pandemic is making me feel out of control. | .628 |  |
| 9 | The corona pandemic is making me feel compassion. |  | .511 |

*Worries Virus and Worries Measures*

Kaiser-Meyer-Olkin Measure of Sampling Adequacy was .84 and Bartlett’s test of sphericity was significant, χ²(df = 55) = 13,097.02, *p* < .001, supporting a rationale for performing PAF with 11 items covering worries. The results indicated two components for items on worries: items on worries about the virus and its effects (*Worries Virus*) and items on worries about the effects of the measures taken to manage the pandemic (*Worries Measures*). The item ‘I worry about society becoming increasingly egoistic’ was excluded because this item had a low Component load on both components. This resulted in two nodes: the node *Worries Virus* that consisted of item 1 to 4 shown below, and the node *Worries Measures* that consisted of items 5 to 10.

| No. | Survey items | Worries Virus Component load | Worries Measures  Component  load |
| --- | --- | --- | --- |
| 1 | I worry about getting infected with the coronavirus. | .718 |  |
| 2 | I worry about infecting others with the coronavirus. | .511 |  |
| 3 | I worry about losing someone I love. | .695 |  |
| 4 | I worry about the health care system overloading. | .627 |  |
| 5 | I worry about what staying at home a lot will do to my health. |  | .716 |
| 6 | I worry about the schools closing. |  | .326 |
| 7 | I worry about a recession. |  | .301 |
| 8 | I worry about limited access to food. |  | .384 |
| 9 | I worry about losing my job. |  | .427 |
| 10 | I worry about getting lonely. |  | .633 |
| *11* | *I worry about society becoming increasingly egoistic.* | *.269* | *.218* |

*Measures Support and Measures Ease*

Kaiser-Meyer-Olkin value was .90 and Bartlett’s test of sphericity was significant, χ²(df = 45) = 31,141.32, *p* < .001, supporting a rationale for performing PAF with the 10 items on attitudes toward the corona measures. The item ‘I think the corona measures will prevent the spread of the coronavirus’ initially loaded on a third component, but due to the low eigenvalue for this component (1.003) we reran the PAF with two fixed factors, resulting in this item loading on the component *Measures Support*. Furthermore, Cronbach’s alpha of the node *Measures Support* did not require the exclusion this item (*a =* .90, if item deleted *a* = .91). The item ‘I find the corona measures... Insufficient - Overdone’ was excluded, because this item has a low Component load on the *Measures Support* component. This resulted in two nodes: the node *Measures Support,* consisting of the items 1 to 7 shown below, and the node *Measures Ease,* consisting of the items 8 and 9.

| No. | Survey items | Measures Support  Component load | Measures Ease  Component load |
| --- | --- | --- | --- |
| 1 | I find the corona measures... Senseless – Sensible. | .869 |  |
| 2 | I find the corona measures... Useless – Useful. | .877 |  |
| 3 | I find the corona measures... Unnecessary – Necessary. | .901 |  |
| 4 | I find the corona measures... Unfair – Fair. | .647 |  |
| 5 | I find the corona measures... Unacceptable – Acceptable. | .776 |  |
| 6 | I think the corona measures will prevent the spread of the coronavirus. | .497 |  |
| 7 | As a society, we must do whatever is necessary to prevent the spread of the coronavirus. | .654 |  |
| 8 | I find the corona measures... Unpleasant – Pleasant. |  | .661 |
| 9 | I find the corona measures... Difficult – Easy. |  | .774 |
| *10* | *I find the corona measures... Insufficient – Overdone.* | *-.242* |  |

*Involvement*

Kaiser-Meyer-Olkin value was .73 and Bartlett’s test of sphericity was significant, χ²(df = 6) = 7,774.14, *p* < .001, supporting a rationale for performing PAF with the four items on the degree to which participants are involved in the corona pandemic. The items loaded on one component, resulting in the node Involvement with the items 1 to 3 shown below. The item ‘To what extent do you feel you can avoid the news about the corona pandemic?’ was excluded, because this item has a low component load on the *Involvement* component.

| No. | Survey items | Involvement Component load |
| --- | --- | --- |
| 1 | How important is the topic of corona pandemic to you? | .832 |
| 2 | To what extent does the news about the corona pandemic have your attention? | .807 |
| 3 | How much do you think about the corona pandemic? | .754 |
| *4* | *To what extent do you feel you can avoid the news about the corona pandemic?* | *-.176* |

*Trust*

Kaiser-Meyer-Olkin value was .73 and Bartlett’s test of sphericity was significant, χ²(df = 6) = 14,759.76, *p* < .001, supporting a rationale for performing PAF with the four items on trust in actor relevant to manage the corona pandemic. The results suggested a single component for the items on trust, resulting in the node *Trust* with the four items shown below.

| No. | Survey items | Trust  Component load |
| --- | --- | --- |
| 1 | I trust the authorities to adequately manage the corona pandemic. | .883 |
| 2 | I trust RIVM during the corona pandemic. | .901 |
| 3 | I trust health care professionals during the corona pandemic. | .543 |
| 4 | I trust science during the corona pandemic. | .767 |

*Healthy Lifestyle*

Healthy Lifestyle was predetermined through operationalization with basic items on diet, exercise and sleep.^[[1]](#footnote-1)^ Kaiser-Meyer-Olkin value was .60 and Bartlett’s test of sphericity was significant, χ²(df = 3) = 1,171.04, *p* < .001, not convincingly supporting a rationale for performing PAF with the three items on healthy lifestyle change compared to before the corona pandemic. Nevertheless, in accordance with the predetermined operationalization the results suggest a single component for the items. This is confirmed by the result that scale reliability statistics (*a* = .49) decrease when excluding items. This resulted in the node *Healthy* *Lifestyle* consisting of the three items shown below.

| No. | Survey items | Healthy Lifestyle Component load |
| --- | --- | --- |
| 1 | I've been eating in the past two weeks, compared to before the corona pandemic...  Less healthy – healthier. | .607 |
| 2 | I've been exercising in the past two weeks, compared to before the corona pandemic… Less – More. | .521 |
| 3 | I've been sleeping for the past two weeks, compared to before the corona pandemic... Worse – Better. | .404 |

## 1.4 Comparing results from intervention conditions

The interventions featured conditions aimed at both increasing and decreasing scores on the same nodes. Consequently, there were no significant differences between scores on nodes that were targeted by the interventions depending on whether participants were assigned to an intervention or control condition (see below).

The table on the next page provides the necessary Mann-Whitney test parameters to compare the scores on the nodes between the conditions participants were randomly assigned to for the interventions. Wave 3 and Wave 5 both contained five conditions: two conditions aimed at increasing and decreasing a central node, two conditions aimed at increasing and decreasing a peripheral node, and a control condition. The following table shows the results of comparing the scores on the nodes of respondents in the control condition with respondents in either of the four intervention condition. The scores on the nodes did not differ significantly depending on whether participants were assigned to one of the intervention conditions or the control condition. The only exception was *Vaccination Intention* in Wave 5: there was a significant difference between the control condition and the combined intervention conditions for this node. The interventions in this wave were aimed at the nodes *Measures Support* and *Economic Consequences*, therefore the interventions do not provide a plausible explanation for this difference.

| Nodes^1^ | Wave 3 |  |  |  | Wave 5 |  |  |  |
| --- | --- | --- | --- | --- | --- | --- | --- | --- |
|  | *Mdn* Control condition  (*n* = 750) | *Mdn* Intervention condition  (*n* = 3,004) | *U* | *p-*value | *Mdn* Control condition  (*n* = 488) | *Mdn* Intervention condition  (*n* = 1,961) | *U* | *p-*value |
| Compliance | 6 | 6 | 1,165,498.50 | .14 | 5.83 | 5.83 | 458,036.00 | .14 |
| Risk Perception | 16 | 16 | 1,150,719.50 | .36 | 15 | 15 | 485,290.00 | .63 |
| Health Risk | 4 | 4 | 1,145,925.00 | .46 | 4 | 4 | 464,738.00 | .32 |
| Economic Consequences | 4 | 4 | 1,133,674.00 | .79 | 3.50 | 3.50 | 479,542.50 | .94 |
| Self-exempting Beliefs | 1.50 | 1.50 | 1,108,881.00 | .49 | 1.50 | 1.50 | 481,802.50 | .81 |
| Negative Affect | 3.50 | 3.38 | 1,100,315.00 | .32 | 3.13 | 3.13 | 478,979.00 | .97 |
| Compassion | 5 | 5 | 1,115,263.50 | .67 | 5 | 5 | 465,307.00 | .34 |
| Worries Virus | 4 | 4 | 1,104,672.00 | .41 | 3.75 | 3.75 | 466,229.50 | .38 |
| Worries Measures | 3 | 3 | 1,142,642.50 | .54 | 2.83 | 2.83 | 471,742.50 | .63 |
| Vaccination Intention | 6 | 6 | 1,145,813.50 | .44 | 7 | 7 | 447,726.50 | .021* |
| Measures Support | 5.71 | 5.71 | 1,129,523.00 | .91 | 5.71 | 5.71 | 462,268.50 | .25 |
| Measures Ease | 4 | 4 | 1,151,451.50 | .34 | 4 | 4 | 461,460.50 | .22 |
| Social Norm | 5 | 5 | 1,139,130.50 | .63 | 4.50 | 4.50 | 457,933.00 | .14 |
| Control Infection | 4.50 | 4.50 | 1,161,302.00 | .18 | 4.50 | 4.50 | 463,272.00 | .27 |
| Self-efficacy | 6 | 6 | 1,158,188.50 | .21 | 6 | 6 | 468,057.50 | .44 |
| Involvement | 5 | 4.67 | 1,091,875.50 | .19 | 4.67 | 4.67 | 468,680.50 | .48 |
| Perceived Knowledge | 5 | 5 | 1,111,568.00 | .56 | 5 | 5 | 454,054.50 | .07 |
| Trust | 5.75 | 5.75 | 1,158,710.00 | .22 | 6 | 6 | 453,831.50 | .08 |
| Health General | 6 | 6 | 1,125,179.50 | .96 | 5 | 5 | 489,679.50 | .40 |
| Health change Physical | 4 | 4 | 1,134,294.50 | .71 | 4 | 4 | 490,732.50 | .27 |
| Health change Mental | 4 | 4 | 1,149,268.50 | .31 | 4 | 4 | 475,838.50 | .82 |
| Healthy Lifestyle | 4 | 4 | 1,134,066.50 | .77 | 4 | 4 | 497,116.50 | .16 |
| Mental Wellbeing~ | 24.11 | 25.03 | 1,163,825.50 | .16 | 25.03 | 25.03 | 464,135.50 | .30 |
| Loneliness~ | 2.17 | 2.17 | 1,110,859.50 | .56 | 2 | 2 | 503,637.00 | .07 |
| Somatic Complaints~ | 1 | 1 | 1,129,146.50 | .91 | 1 | 1 | 482,149.00 | .77 |
| Depressive Complaints~ | 1.50 | 1.50 | 1,095,845.00 | .24 | 1.33 | 1.33 | 482,215.50 | .78 |
| Anxiety Complaints~ | 1.33 | 1.33 | 1,088,371.00 | .14 | 1.17 | 1.17 | 484,616.00 | .65 |

^1^ 7-point Likert-scale, unless marked ~, indicating a 5-point Likert-scale. Please note that *Risk* *Perception* is the product of two items and *Mental* *Wellbeing* is the metric sum score of all items.

* Significant difference (*p* < .05)

## 1.5 R script

See OSF (<https://osf.io/qu7p2/?view_only=01a4c512371c460cab4919f2dcbb7bdc>) for the .R file containing the R script (R Core Team, 2013). Besides the values specified in the R-script, the default values were used.

The script and the interpretation of the output was based on the tutorial paper of (Epskamp, 2020a). We used the package *qgraph* (Epskamp et al., 2012) to visualize the graphs, *bootnet* (Epskamp et al., 2018) for the stability and accuracy measures and *psychonetrics* (Epskamp, 2020b) for the temporal analyses.

Please note that the layout of the network (i.e., position of the nodes) was determined by the average layout of both the temporal and contemporaneous networks. Subsequently, we manually adjusted the position of several nodes to improve readability, as specified in the script.

# Results

## 2.1 Descriptive statistics and Repeated Measures ANOVA

The statistics relevant for the temporal dynamics of the COVID-19 broad attitude networks are presented in the table below (i.e., longitudinal sample of participants that completed all waves). The table also includes the results of a repeated measures ANOVA to compare the mean scores on the continues variables for the different waves (i.e., T1 – T5; see column T5), which is discussed below.

The Kolmogorov-Smirnov value was significant for all variables (*p* < .001). Given the large sample size, we based the decisions concerning data transformation on the QQ-plots. The variables *Health Risk, Economic Consequences, Negative Affect, Worries Virus* and *Measures Ease* were treated as normally distributed and therefore not transformed. The rest of the variables were transformed with log10(x), unless negatively skewed, in which case we used the log10(k-x) transformation. The latter applied to the following variables: *Compliance, Compassion, Vaccination Intention, Measures Support, Social Norm, Control Infection, Self-efficacy, Involvement, Perceived Knowledge, Trust* and *Health General.* When the assumption of sphericity was violated, we report the lower-bound corrected results by default, unless otherwise specified. Post hoc analysis for all nodes was based on estimated marginal means with a Bonferroni adjustment for multiple comparisons.

The results indicate a significant effect of time on all nodes. A detailed description of the repeated measures ANOVA results and post hoc analysis with a specification of which waves significantly differ is provided below. *Compliance* with behavioural measures significantly decreased over time. All nodes on elements of attitudes relevant for the pandemic and the behavioural measures (e.g., perceived [health] risk, negative affect, worries and support for the measures) decreased in average score over time, with the exception of *Measures Ease*. With regard to the additional psychological nodes, the scores on *Social Norm* and *Involvement* decreased, whereas scores for *Control Infection* increased. *Trust* initially decreased, but showed an increase between the last two measurements. Participants reported an improvement in the health-related nodes: the majority of the health-related nodes decreased over time, with the exception of *Health change Mental*, *Healthy* *Lifestyle* and *Mental Wellbeing* that increased over time. The largest effect sizes were found for the nodes *Compliance* (ƞ² = .184), *Worries Virus* (ƞ² = .120), *Social* *Norm* (ƞ² = .109) and *Involvement* (ƞ² = .103), which all decreased over time.

| Completed waves | Wave 1 - 5 | | | | |
| --- | --- | --- | --- | --- | --- |
|  | T1 | T2 | T3 | T4 | T5 |
| Nodes^1^ | M (SD) | M (SD) | M (SD) | M (SD) | M (SD) |
| Compliance | 6.11 (0.77) | 6.10 (0.77) | 5.92 (0.87) | 5.79 (0.93) | 5.68 (1)** |
| Risk Perception | 18.35 (8.81) | 17.88 (8.76) | 17.39 (8.8) | 17.09 (8.76) | 16.41 (9.03)** |
| Health Risk | 4.25 (1.12) | 4.24 (1.15) | 4.18 (1.2) | 4.17 (1.22) | 4.11 (1.27)** |
| Economic Consequences | 3.99 (1.37) | 3.79 (1.33) | 3.73 (1.35) | 3.65 (1.36) | 3.48 (1.33)** |
| Self-exempting Beliefs | 2.04 (1.10) | 1.99 (1.10) | 1.95 (1.09) | 1.99 (1.12) | 1.92 (1.09)** |
| Negative Affect | 3.63 (1.26) | 3.58 (1.31) | 3.40 (1.35) | 3.28 (1.41) | 3.12 (1.43)** |
| Compassion | 5.14 (1.35) | 5 (1.41) | 4.80 (1.46) | 4.64 (1.49) | 4.52 (1.53)** |
| Worries Virus | 4.31 (1.17) | 4.17 (1.18) | 3.96 (1.20) | 3.91 (1.22) | 3.75 (1.25)** |
| Worries Measures | 3.21 (0.99) | 3.15 (1.01) | 3.06 (1.02) | 2.99 (1.03) | 2.84 (1.05)** |
| Vaccination Intention | 5.84 (1.64) | 5.75 (1.73) | 5.58 (1.83) | 5.50 (1.86) | 5.38 (1.95)** |
| Measures Support | 5.74 (1.03) | 5.59 (1.06) | 5.51 (1.16) | 5.36 (1.21) | 5.40 (1.27)** |
| Measures Ease | 3.72 (1.29) | 3.81 (1.28) | 3.85 (1.28) | 3.84 (1.28) | 3.92 (1.32)** |
| Social Norm | 5.14 (1.06) | 4.85 (1.10) | 4.77 (1.15) | 4.55 (1.23) | 4.38 (1.27)** |
| Control Infection | 4.52 (1.07) | 4.50 (1.04) | 4.51 (1.02) | 4.52 (1.01) | 4.57 (1.05)* |
| Self-efficacy | 5.61 (1.18) | 5.59 (1.13) | 5.60 (1.12) | 5.55 (1.12) | 5.59 (1.11)* |
| Involvement | 5.07 (1.15) | 4.89 (1.19) | 4.76 (1.23) | 4.56 (1.27) | 4.55 (1.29)** |
| Perceived Knowledge | 4.77 (1.17) | 4.75 (1.15) | 4.76 (1.12) | 4.78 (1.11) | 4.80 (1.15)* |
| Trust | 5.63 (1.07) | 5.50 (1.08) | 5.52 (1.12) | 5.42 (1.15) | 5.50 (1.16)** |
| Health General | 5.30 (1.28) | 5.28 (1.27) | 5.25 (1.24) | 5.24 (1.25) | 5.23 (1.25)** |
| Health change Physical | 4.14 (0.89) | 4.04 (0.82) | 4.01 (0.82) | 4.01 (0.81) | 4 (0.84)** |
| Health change Mental | 3.88 (0.89) | 3.87 (0.87) | 3.87 (0.86) | 3.89 (0.84) | 3.94 (0.86)* |
| Healthy Lifestyle | 3.86 (0.74) | 3.85 (0.73) | 3.89 (0.71) | 3.89 (0.66) | 3.91 (0.68)* |
| Mental Wellbeing~ | 24.45 (4.13) | 24.64 (4.29) | 24.78 (4.36) | 24.92 (4.56) | 25.26 (4.58)** |
| Loneliness~ | 2.32 (0.75) | 2.30 (0.77) | 2.26 (0.78) | 2.22 (0.81) | 2.16 (0.81)** |
| Somatic Complaints~ | 1.36 (0.54) | 1.33 (0.52) | 1.31 (0.53) | 1.30 (0.54) | 1.30 (0.54)** |
| Depressive Complaints~ | 1.90 (0.90) | 1.86 (0.91) | 1.80 (0.91) | 1.77 (0.92) | 1.69 (0.9)** |
| Anxiety Complaints~ | 1.77 (0.87) | 1.73 (0.86) | 1.67 (0.85) | 1.65 (0.86) | 1.58 (0.83)** |

^1^ 7-point Likert-scale, unless marked ~, indicating a 5-point Likert-scale. Please note that *Risk* *Perception* is the product of two items and *Mental* *Wellbeing* is the sum score of all items, after which raw scores were converted to metric scores.
* *p* < .05; ** *p* <.001; indicating a significant effect of time on the node. A detailed description of the repeated measures ANOVA results and post hoc analysis with a specification of which waves significantly differ is provided below.

*Compliance*

Mauchly’s test indicated that the assumption of sphericity had been violated, $\chi^{2}$(9) = 776.46, *p* <.001. The lower-bound corrected results showed that there was a significant effect of time on *Compliance*, *F*(1, 2448) = 553.41, *p* <.001, ƞ² = .184. Post hoc analysis showed that with the exception of T1 with T2 (*p* = 1.00), all pairwise comparisons differed significantly, *p* <.001. These results suggest that the degree to which participants indicated to comply with the behavioural measures decreased over time.

*Risk Perception*

Mauchly’s test indicated a violation of the sphericity assumption, $\chi^{2}$(9) = 330.08, *p* <.001. There was a statistically significant effect of time on *Risk Perception*, *F*(1, 2448) = 75.75, *p* <.001, ƞ² = .030. Post hoc analysis showed that with the exception of T3 with T4 (*p* = .084), all pairwise comparisons differed significantly (*p*-values <.001, with one exception of *p* = .037). These results suggest that participants’ perception of the risk of getting infected with the coronavirus decreased over time.

*Health Risk*

Mauchly’s test indicated that the assumption of sphericity had been violated, $\chi^{2}$(9) = 173.94, *p* <.001. The results showed that there was a significant effect of time on *Health Risk*, *F*(1, 2398) = 17.37, *p* <.001, ƞ² = .007. Post hoc analysis showed that with the exception of T1 with T2 (*p* = 1.00) and T3 with T4 (*p* = 1.00), all pairwise comparisons differed significantly (*p*-value ranges from <.001 to .039). These results suggest that participants’ perception of the health risk in case of an infection with the coronavirus decreased over time.

*Economic Consequences*

Mauchly’s test indicated a violation of the sphericity assumption, $\chi^{2}$(9) = 150.09, *p* <.001. There was a statistically significant effect of time on *Economic Consequences*, *F*(1, 2398) = 122.58, *p* <.001, ƞ² = .049. Post hoc analysis showed that with the exception of T2 with T3 (*p* = .52), all pairwise comparisons differed significantly (*p*-value ranges from <.001 to .019). These results suggest that participants’ view on the economic consequences of the pandemic decreased over time.

*Self-exempting Beliefs*

Mauchly’s test indicated that the assumption of sphericity had been violated, $\chi^{2}$(9) = 101.68, *p* <.001. The lower-bound corrected results showed that there was a significant effect of time on *Self-exempting Beliefs*, *F*(1, 2448) = 14.63, *p* <.001, ƞ² = .006. Post hoc analysis showed that the following measurements differed significantly: T1 with T2 (*p* = .031), T1 with T3 (*p* <.001), T1 with T4 (*p* = .007), T1 with T5 (*p* <.001), T2 with T5 (*p* = .001) and T4 with T5 (*p* = .001). These results suggest that participants’ self-exempting beliefs decreased over time.

*Negative Affect*

Mauchly’s test indicated a violation of the sphericity assumption, $\chi^{2}$(9) = 523.03, *p* <.001. There was a statistically significant effect of time on *Negative Affect*, *F*(1, 2398) = 259.65, *p* <.001, ƞ² = .098. Post hoc analysis showed that with the exception of T1 with T2 (*p* = .072), all pairwise comparisons differed significantly, *p* <.001. These results suggest that the degree to which participants felt that the pandemic caused negative emotions decreased over time.

*Compassion*

Mauchly’s test indicated that the assumption of sphericity had been violated, $\chi^{2}$(9) = 108.13, *p* <.001. The lower-bound corrected results showed that there was a significant effect of time on *Compassion*, *F*(1, 2448) = 190.71, *p* <.001, ƞ² = .072. Post hoc analysis showed that all pairwise comparisons differed significantly, *p* <.001. These results suggest that the degree to which the pandemic makes participants feel compassionate decreased over time.

*Worries Virus*

Mauchly’s test indicated a violation of the sphericity assumption, $\chi^{2}$(9) = 293.06, *p* <.001. There was a statistically significant effect of time on *Worries Virus*, *F*(1, 2398) = 328.16, *p* <.001, ƞ² = .120. Post hoc analysis showed that all pairwise comparisons differed significantly (*p*-value ranges from <.001 to .002). These results suggest that the degree to which participants worry about events related to the coronavirus (i.e. getting infected or infecting others, losing a loved one or the health care system getting overloaded) decreased over time.

*Worries Measures*

Mauchly’s test indicated that the assumption of sphericity had been violated, $\chi^{2}$(9) = 304.92, *p* <.001. The lower-bound corrected results showed that there was a significant effect of time on *Worries Measures*, *F*(1, 2448) = 231.89, *p* <.001, ƞ² = .087. Post hoc analysis showed that all pairwise comparisons differed significantly, *p* <.001. These results suggest that the degree to which participants worry about events related to the corona measures (e.g., health effects of staying home, schools closing, a recession) decreased over time.

*Vaccination Intention*

Mauchly’s test indicated a violation of the sphericity assumption, $\chi^{2}$(9) = 883.50, *p* <.001. There was a statistically significant effect of time on *Vaccination Intention*, *F*(1, 2448) = 118.27, *p* <.001, ƞ² = .046. Post hoc analysis showed that all pairwise comparisons differed significantly (*p*-values <.001, with one exception of *p* = .009). These results suggest that participants intention to get vaccinated (if available) decreased over time.

*Measures Support*

Mauchly’s test indicated that the assumption of sphericity had been violated, $\chi^{2}$(9) = 507.60, *p* <.001. The lower-bound corrected results showed that there was a significant effect of time on *Measures Support*, *F*(1, 2448) = 172.94, *p* <.001, ƞ² = .066. Post hoc analysis showed that all pairwise comparisons differed significantly, *p* <.001. These results suggest that participants’ support for the corona measures decreased over time.

*Measures Ease*

Mauchly’s test indicated a violation of the sphericity assumption, $\chi^{2}$(9) = 294.16, *p* <.001. There was a statistically significant effect of time on *Measures Ease*, *F*(1, 2398) = 24.25, *p* <.001, ƞ² = .010. Post hoc analysis showed that with the exception of T2 with T3 (*p* = .429), T2 with T4 (*p* = 1.00) and T3 with T4 (*p* = 1.00), all pairwise comparisons differed significantly (*p*-value ranges from <.001 to .004). These results suggest that participants’ perceived ease of the corona measures increased over time (i.e. participants experienced the corona measures as easier at the end of the data collection compared to the first survey).

*Social Norm*

Mauchly’s test indicated that the assumption of sphericity had been violated, $\chi^{2}$(9) = 102.79, *p* <.001. The lower-bound corrected results showed that there was a significant effect of time on *Social Norm*, *F*(1, 2448) = 298.08, *p* <.001, ƞ² = .109. Post hoc analysis showed that all pairwise comparisons differed significantly (*p*-values <.001, with one exception of *p* = .022). These results suggest that the degree to which participants think other people (find it important to) comply with the corona measures decreased over time.

*Control Infection*

Mauchly’s test indicated a violation of the sphericity assumption, $\chi^{2}$(9) = 73.46, *p* <.001. There was a statistically significant effect of time on *Control Infection*, *F*(1, 2448) = 3.88, *p* = .049, ƞ² = .002. Post hoc analysis showed that T2 differed significantly from T5 (*p* = .010), T3 differed significantly from T5 (*p* = .009) and T4 differed significantly from T5 (*p* = .022). The remaining pairwise comparisons did not differ significantly (*p*-value ranges from .536 to 1.00). These result suggest an increase in participants’ perceived control to avoid an infection with the coronavirus between the middle and last surveys.

*Self-efficacy*

Mauchly’s test indicated that the assumption of sphericity had been violated, $\chi^{2}$(9) = 147.67, *p* <.001. The lower-bound corrected results showed that there was a significant effect of time on *Self-efficacy*, *F*(1, 2448) = 5,43, *p* = .020, ƞ² = .002. Post hoc analysis showed that the following measurements differed significantly: T1 with T4 (*p* <.001), T2 with T4 (*p* = .035) and T3 with T4 (*p* = .047). The remaining pairwise comparisons did not differ significantly (*p*-value ranges from .202 to 1.00). These result suggest a decrease over time in participants’ perceived ability to protect themselves, more specifically between the first three waves and the forth wave.

*Involvement*

Mauchly’s test indicated a violation of the sphericity assumption, $\chi^{2}$(9) = 368.75, *p* <.001. There was a statistically significant effect of time on *Involvement*, *F*(1, 2448) = 280,31, *p* <.001, ƞ² = .103. Post hoc analysis showed that with the exception of T4 with T5 (*p* = 1.00), all pairwise comparisons differed significantly (*p* <.001). These result suggest that the degree to which participants are involved in the corona pandemic decreased over time.

*Perceived Knowledge*

Mauchly’s test indicated that the assumption of sphericity had been violated, $\chi^{2}$(9) = 157.43, *p* <.001. Since sphericity is violated (Ɛ = .97) and the lower-bound corrected were not significant, Huynh-Feldt corrected results are reported^[[2]](#footnote-2)^. The results showed that there was a significant effect of time on *Perceived Knowledge*, *F*(3.88, 9487.88) = 3.23, *p* = .013, ƞ² = .001. Post hoc analysis showed that T2 and T3 differed significantly from T5 (*p* = .016 and *p* = .023, respectively). The remaining pairwise comparisons did not differ significantly (*p*-value ranges from .457 to 1.00). These result suggest an increase over time in participants’ perceived knowledge about the corona pandemic.

*Trust*

Mauchly’s test indicated a violation of the sphericity assumption, $\chi^{2}$(9) = 198.43, *p* <.001. There was a statistically significant effect of time on *Trust*, *F*(1, 2448) = 49.49, *p* <.001, ƞ² = .020. Post hoc analysis showed that with the exception of T2 with T3 (*p* = .458), T2 with T5 (*p* = .589) and T3 with T5 (*p* = 1.00), all pairwise comparisons differed significantly (*p* <.001). These result suggest an initial decrease over time in participants’ trust in the actors relevant for the pandemic, followed by an increase between the last two surveys.

*Health General*

Mauchly’s test indicated that the assumption of sphericity had been violated, $\chi^{2}$(9) = 193.47, *p* <.001. The lower-bound corrected results showed that there was a significant effect of time on *Health General*, *F*(1, 2448) = 12.54, *p* <.001, ƞ² = .005. Post hoc analysis showed that the following pairwise comparisons differed significantly: T1 with T3 (*p* <.001), T1 with T4 (*p* <.001), T1 with T5 (*p* <.001), T2 with T3 (*p* = .040), T2 with T4 (*p* = .003) and T2 with T5 (*p* = .001). The remaining pairwise comparisons did not differ significantly (*p*-value ranges from .925 to 1.00). These results suggest that participants’ rating of their health in general decreased over time.

*Health change Physical*

Mauchly’s test indicated a violation of the sphericity assumption, $\chi^{2}$(9) = 441.08, *p* <.001. There was a statistically significant effect of time on *Health change Physical*, *F*(1, 2448) = 20.65, *p* <.001, ƞ² = .008. Post hoc analysis showed that T1 differed significantly from all subsequent waves (*p* <.001), whereas the remaining pairwise comparisons did not differ significantly (*p*-value ranges from .126 to 1.00). These results suggest that participants initially experienced an improvement in their physical health compared to before the pandemic, but this improvement disappeared after the first survey. From there on participants viewed their physical health as comparable to their physical health before the pandemic.

*Health change Mental*

Mauchly’s test indicated that the assumption of sphericity had been violated, $\chi^{2}$(9) = 378.64, *p* <.001. The lower-bound corrected results showed that there was a significant effect of time on *Health change Mental*, *F*(1, 2448) = 5.99, *p* = .014, ƞ² = .002. Post hoc analysis showed that all waves differed significantly from T5 (*p*-value ranges from <.001 to .018), whereas the remaining pairwise comparisons did not differ significantly (*p* = 1.00). These results suggest that participants initially experienced a deterioration in their mental health compared to before the pandemic. Although they reported a deterioration in all surveys, the experienced deterioration in mental health compared to before the pandemic decreased in the last survey.

*Healthy Lifestyle*

Mauchly’s test indicated a violation of the sphericity assumption, $\chi^{2}$(9) = 517.18, *p* <.001. There was a statistically significant effect of time on *Healthy Lifestyle*, *F*(1, 2448) = 8.86, *p* = .003, ƞ² = .004. Post hoc analysis showed that the following pairwise comparisons differed significantly: T1 with T4 (*p* = .032), T1 with T5 (*p* = .013), T2 with T3 (*p* = .008), T2 with T4 (*p* <.001) and T2 with T5 (*p* < .001). The remaining pairwise comparisons did not differ significantly (*p*-value ranges from .493 to 1.00). These results suggest that participants initially experienced a decrease in their healthy lifestyle compared to before the pandemic. Although they reported a decrease in all surveys, the experienced decrease in healthy lifestyle compared to before the pandemic lessened over time.

*Mental Wellbeing*

Mauchly’s test indicated that the assumption of sphericity had been violated, $\chi^{2}$(9) = 137.26, *p* <.001. The lower-bound corrected results showed that there was a significant effect of time on *Mental Wellbeing*, *F*(1, 2448) = 37.10, *p* <.001, ƞ² = .015. Post hoc analysis showed that with the exception of T1 and T2 (*p* = .265), T2 and T3 (*p* = .299) and T3 with T4 (*p* = . 666), all pairwise comparisons differed significantly (*p*-value ranges from <.001 to .001). These results suggest that participants’ mental wellbeing improved between the start and the end of the study.

*Loneliness*

Mauchly’s test indicated a violation of the sphericity assumption, $\chi^{2}$(9) = 246.19, *p* <.001. There was a statistically significant effect of time on *Loneliness*, *F*(1, 2448) = 100.32, *p* <.001, ƞ² = .039. Post hoc analysis showed that with the exception of T1 with T2 (*p* = .054), all pairwise comparisons differed significantly (*p* <.001). These results suggest that the degree to which participants felt lonely decreased over time.

*Somatic Complaints*

Mauchly’s test indicated that the assumption of sphericity had been violated, $\chi^{2}$(9) = 388.48, *p* <.001. The lower-bound corrected results showed that there was a significant effect of time on *Somatic Complaints*, *F*(1, 2448) = 26.80, *p* <.001, ƞ² = .011. Post hoc analysis showed that T1 differed significantly from all subsequent waves (*p* <.001). Furthermore, the pairwise comparisons T2 with T4 and T2 with T5 differed significantly (*p* = .036 and *p* = .008, respectively), and T3 differed from T5 (*p* = .005). The remaining pairwise comparisons did not differ significantly (*p*-value ranges from .069 to 1.00). These results suggest that the degree to which participants experienced somatic complaints decreased over time.

*Depressive Complaints*

Mauchly’s test indicated a violation of the sphericity assumption, $\chi^{2}$(9) = 511.15, *p* <.001. There was a statistically significant effect of time on *Depressive Complaints*, *F*(1, 2448) = 142.73, *p* <.001, ƞ² = .055. Post hoc analysis showed that all pairwise comparisons differed significantly (*p*-value ranges from <.001 to .002). These results suggest a decrease over time in the degree to which participants experienced depressive complaints.

*Anxiety Complaints*

Mauchly’s test indicated that the assumption of sphericity had been violated, $\chi^{2}$(9) = 419.64, *p* <.001. The lower-bound corrected results showed that there was a significant effect of time on *Anxiety Complaints*, *F*(1, 2448) = 121.32, *p* <.001, ƞ² = .047. Post hoc analysis showed that all pairwise comparisons differed significantly (*p*-value ranges from <.001 to .011). These results suggest that the degree to which participants experienced anxiety complaints decreased over time.

## 2.2 Network analysis model fit evaluation

The values provided below present the model fit of the dlvm1 model of the *psychonetrics* package. We used the dlvm1 model of the *psychonetrics* package to estimate the model. We specified within latent (ggm [Gaussian graphical model]), between latent (chol [Cholesky]), within and between residuals (cov [covariance]), and estimator (FIML). Overall fit, including the temporal and contemporaneous networks, showed excellent fit according to RMSEA (root mean square error of approximation = .023 [95% CI 0.023–0.024]) and CFI (comparative fit index = .96), although the standard chi-square test, not accounting for the freedom, rejected the model, χ²(7803) = 17930.86, p < .001. See table on the next page for additional measures.

| Measure | Value |
| --- | --- |
| logl | -373328.903 |
| unrestricted.logl | -364363.473 |
| baseline.logl | -499487.446 |
| nvar | 135 |
| nobs | 9315 |
| npar | 1512 |
| df | 7803 |
| objective | 63.124 |
| chisq | 17930.859 |
| pvalue | 0.000 |
| baseline.chisq | 270247.946 |
| baseline.df | 9045 |
| baseline.pvalue | 0.000 |
| nfi | 0.934 |
| pnfi | 0.805 |
| tli | 0.955 |
| nnfi | 0.955 |
| rfi | 0.923 |
| ifi | 0.961 |
| rni | 0.961 |
| cfi | 0.961 |
| rmsea | 0.023 |
| rmsea.ci.lower | 0.023 |
| rmsea.ci.upper | 0.024 |
| rmsea.pvalue | 1.000 |
| aic.ll | 749681.805 |
| aic.ll2 | 754845.814 |
| aic.x | 2324.859 |
| aic.x2 | 20954.859 |
| bic | 758425.410 |
| bic2 | 753621.452 |
| ebic.25 | 765842.185 |
| ebic.5 | 773258.961 |
| ebic.75 | 779192.381 |
| ebic1 | 788092.512 |

## 2.3 COVID-19 broad attitude networks including all edges


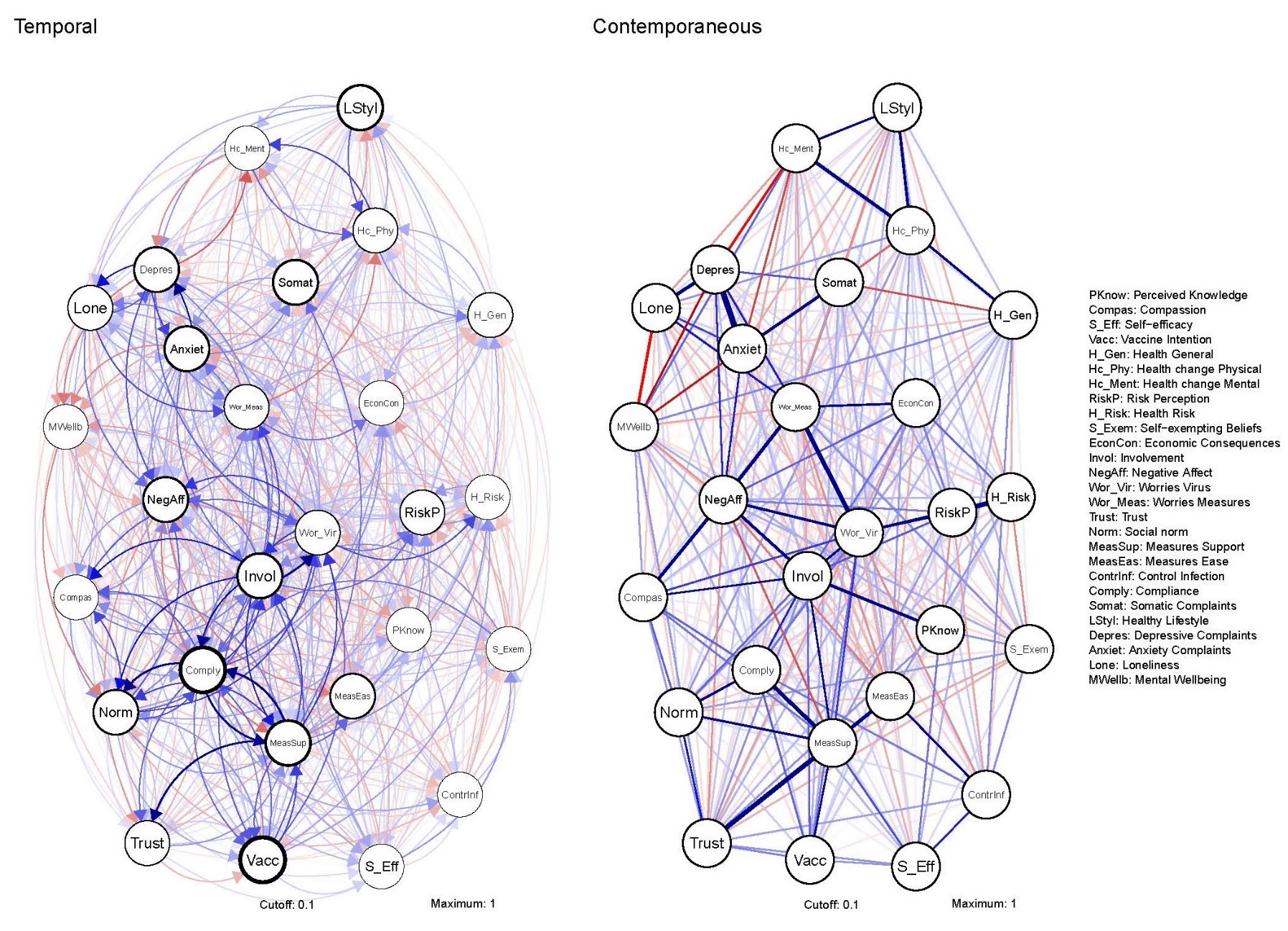


## 2.4 Edge weights and node strength of COVID-19 broad attitude networks

**Temporal COVID-19 network**

Read rows (first column) as node from which the edge originates. Please note that the value .00 in the table does not indicate an edge was absent, because these values were rounded to two decimals.

| To  From | Perceived knowledge | Compassion | Self-efficacy | Vaccine Intention | Health General | Health change Physical | Health change Mental | Risk Perception | Health Risk | Self-exempting Beliefs | Economic Consequences | Involvement | Negative Affect | Worries Virus | Worries Measures | Trust | Social norm | Measures Support | Measures Ease | Control Infection | Compliance | Somatic Complaints | Healthy Lifestyle | Depressive Complaints | Anxiety Complaints | Loneliness | Mental Well-being |
| --- | --- | --- | --- | --- | --- | --- | --- | --- | --- | --- | --- | --- | --- | --- | --- | --- | --- | --- | --- | --- | --- | --- | --- | --- | --- | --- | --- |
| Perceived knowledge | .09 | .00 | .02 | -.01 | .01 | .01 | -.01 | -.01 | -.02 | -.02 | -.04 | -.02 | -.02 | -.03 | -.02 | .01 | -.04 | .01 | .03 | .02 | -.01 | .00 | .02 | -.02 | -.03 | -.01 | .01 |
| Compassion | -.01 | .10 | .00 | .00 | .01 | .00 | -.02 | .00 | .01 | .01 | .02 | .05 | .05 | .03 | .01 | .01 | .04 | .01 | -.02 | .02 | .04 | -.02 | -.01 | .01 | .01 | .00 | -.02 |
| Self-efficacy | .02 | -.02 | .08 | -.02 | -.02 | -.01 | .01 | -.02 | .00 | .04 | -.01 | -.03 | .02 | -.01 | -.01 | .01 | -.01 | -.01 | .01 | .01 | -.03 | -.01 | .00 | .00 | .01 | .01 | -.01 |
| Vaccine Intention | .00 | .05 | .02 | .41 | -.01 | .01 | .00 | .04 | .04 | -.01 | .02 | .06 | .03 | .06 | .01 | .02 | .04 | .08 | .01 | .00 | .06 | .01 | -.01 | .01 | .03 | .00 | .01 |
| Health General | -.01 | .01 | -.01 | .01 | .15 | .03 | .00 | .01 | .01 | .00 | .03 | .02 | .02 | .01 | .02 | .02 | .03 | .00 | -.02 | -.01 | .00 | .00 | .01 | .01 | .00 | .02 | .00 |
| Health change Physical | .02 | .02 | .04 | .02 | .05 | .14 | .08 | .01 | .01 | .02 | .02 | .02 | .03 | .02 | .00 | -.01 | .02 | .04 | .00 | .01 | .02 | -.02 | .04 | -.01 | .00 | .00 | -.01 |
| Health change Mental | -.04 | .00 | -.01 | .00 | .01 | .06 | .10 | .00 | .02 | .00 | .00 | -.01 | -.02 | .01 | -.01 | .00 | .00 | -.01 | -.01 | .00 | -.01 | .03 | .01 | -.05 | -.03 | -.02 | .02 |
| Risk Perception | .01 | .02 | -.01 | .00 | -.01 | .02 | -.01 | .11 | .04 | -.02 | .00 | .03 | .01 | .02 | .02 | .00 | .02 | .00 | -.02 | -.02 | .01 | .03 | .03 | .00 | .00 | .02 | .00 |
| Health Risk | -.03 | .00 | .01 | -.02 | -.01 | -.01 | .02 | .04 | .09 | -.02 | .02 | -.02 | -.02 | .02 | -.02 | .02 | -.03 | .02 | .03 | -.01 | .00 | -.01 | -.02 | .00 | -.01 | -.02 | .00 |
| Self-exempting Beliefs | -.02 | .01 | .02 | .03 | -.01 | .01 | .01 | -.03 | -.02 | .06 | .01 | .04 | .00 | .00 | .01 | .01 | .02 | .02 | .01 | .02 | .06 | -.02 | -.01 | -.03 | -.03 | .00 | .01 |
| Economic Consequences | .00 | .00 | .03 | .01 | .03 | .02 | -.01 | .00 | .03 | .01 | .10 | .00 | .01 | .02 | .03 | -.01 | -.01 | .00 | -.01 | .02 | .02 | -.02 | -.02 | -.02 | .00 | .00 | -.03 |
| Involvement | .03 | .09 | .00 | .07 | .01 | .01 | -.04 | .06 | .03 | .01 | .02 | .21 | .06 | .11 | .07 | .00 | .10 | .09 | -.01 | .00 | .10 | .01 | -.02 | .05 | .05 | .02 | -.04 |
| Negative Affect | -.03 | .04 | .00 | -.01 | .02 | .01 | -.01 | .00 | .00 | .02 | .00 | .03 | .19 | .03 | .05 | -.04 | -.02 | -.06 | -.05 | -.02 | -.03 | .00 | .02 | .03 | .01 | .02 | -.01 |
| Worries Virus | .00 | .07 | .00 | .02 | -.02 | -.01 | .00 | .06 | .07 | .00 | .04 | .08 | .08 | .13 | .03 | .03 | .03 | .04 | .00 | -.01 | .06 | .01 | -.02 | .04 | .03 | .04 | -.01 |
| Worries Measures | .01 | .01 | -.02 | .00 | -.02 | -.05 | .00 | .03 | -.02 | .01 | .06 | .02 | .02 | .04 | .12 | .01 | .01 | .00 | -.02 | .00 | .01 | .01 | -.02 | .02 | .00 | .05 | -.02 |
| Trust | .02 | -.01 | .00 | -.03 | .02 | -.02 | .02 | .01 | .00 | -.01 | -.03 | -.02 | -.03 | .00 | -.01 | .11 | -.03 | .04 | .00 | .01 | .01 | -.02 | .01 | .00 | .00 | .00 | -.02 |
| Social norm | .00 | .07 | .02 | .03 | -.01 | .01 | -.01 | .04 | .02 | .02 | .04 | .06 | .06 | .08 | .05 | .01 | .16 | .04 | -.02 | -.03 | .07 | .01 | -.01 | .02 | .03 | .02 | -.02 |
| Measures Support | -.01 | .05 | .02 | .07 | .04 | .02 | .00 | .04 | .04 | -.02 | .03 | .08 | .02 | .08 | .03 | .11 | .08 | .25 | .06 | -.02 | .13 | .06 | .00 | .02 | .03 | .01 | .01 |
| Measures Ease | .03 | -.01 | .02 | -.01 | -.02 | .02 | .02 | .00 | .02 | -.01 | -.02 | .00 | -.04 | -.02 | -.03 | .01 | -.01 | .02 | .16 | .01 | .00 | -.01 | .00 | -.02 | -.03 | -.01 | .01 |
| Control Infection | .00 | .00 | .02 | .01 | .01 | -.02 | .00 | -.03 | -.02 | .02 | .01 | -.01 | -.01 | -.01 | .00 | .00 | .00 | -.03 | -.01 | .04 | -.03 | -.03 | .00 | .01 | .01 | .00 | .03 |
| Compliance | .00 | .07 | .03 | .06 | .00 | .01 | -.01 | .02 | .02 | .00 | .04 | .08 | .03 | .07 | .06 | .06 | .12 | .11 | .00 | .04 | .37 | .01 | .01 | .03 | .03 | .04 | -.01 |
| Somatic Complaints | -.01 | -.02 | -.01 | .01 | -.03 | -.03 | .03 | .02 | .01 | .02 | .00 | -.01 | .01 | .01 | .01 | -.02 | .00 | .02 | .00 | -.02 | -.01 | .21 | -.04 | .03 | .04 | .02 | -.01 |
| Healthy Lifestyle | .02 | .02 | .02 | .00 | .02 | .07 | .03 | .01 | .00 | .01 | -.02 | .00 | .02 | -.01 | -.01 | .00 | -.01 | -.01 | .00 | .00 | .02 | -.02 | .24 | .04 | .01 | .03 | .02 |
| Depressive Complaints | .00 | .01 | .03 | .01 | .02 | .00 | -.07 | .01 | .01 | -.02 | .02 | .03 | .07 | .06 | .05 | .00 | -.01 | .01 | -.01 | .00 | -.01 | .02 | .00 | .18 | .07 | .09 | -.05 |
| Anxiety Complaints | .00 | .01 | -.03 | .03 | -.03 | .01 | -.03 | .02 | .02 | -.02 | .02 | .06 | .04 | .04 | .04 | .02 | .03 | .03 | .00 | -.03 | .04 | .05 | -.02 | .10 | .20 | .02 | -.05 |
| Loneliness | -.02 | -.01 | -.01 | .00 | .00 | .03 | -.01 | .01 | .01 | -.01 | .02 | .01 | .06 | .02 | .07 | -.02 | .00 | .00 | -.03 | -.01 | .03 | .03 | .01 | .06 | .03 | .14 | -.04 |
| Mental Well-being | .00 | -.03 | .00 | .01 | -.01 | .00 | .01 | .01 | -.02 | .01 | -.01 | -.03 | -.03 | -.02 | -.03 | .01 | -.05 | .00 | -.02 | .01 | -.03 | .02 | .00 | -.02 | .00 | -.02 | .09 |

| **Contemporaneous  COVID-19 network** | Perceived knowledge | Compassion | Self-efficacy | Vaccine Intention | Health General | Health change Physical | Health change Mental | Risk Perception | Health Risk | Self-exempting Beliefs | Economic Consequences | Involvement | Negative Affect | Worries Virus | Worries Measures | Trust | Social norm | Measures Support | Measures Ease | Control Infection | Compliance | Somatic Complaints | Healthy Lifestyle | Depressive Complaints | Anxiety Complaints | Loneliness | Mental Wellbeing |
| --- | --- | --- | --- | --- | --- | --- | --- | --- | --- | --- | --- | --- | --- | --- | --- | --- | --- | --- | --- | --- | --- | --- | --- | --- | --- | --- | --- |
| Perceived knowledge |  | .01 | .05 | -.02 | .02 | .01 | -.02 | .01 | .02 | .04 | -.01 | .17 | -.02 | -.01 | .00 | .04 | -.02 | .00 | .01 | .02 | -.01 | .00 | .00 | -.02 | -.01 | -.03 | .03 |
| Compassion |  |  | .01 | .00 | .03 | .02 | .02 | -.01 | .00 | .00 | .01 | .10 | .18 | .08 | .02 | .04 | .06 | .05 | -.01 | .00 | .06 | -.01 | -.02 | .00 | .00 | -.02 | .04 |
| Self-efficacy |  |  |  | .04 | .03 | .00 | -.01 | -.04 | .00 | .01 | .00 | .02 | .01 | -.01 | -.03 | .04 | .01 | .07 | .02 | .09 | .03 | -.02 | .00 | .00 | -.01 | .00 | .02 |
| Vaccine Intention |  |  |  |  | .02 | .02 | -.01 | .02 | .02 | .00 | .00 | .06 | .02 | .06 | -.01 | .05 | .02 | .10 | .00 | -.01 | .06 | .03 | .03 | .00 | .00 | .00 | -.01 |
| Health General |  |  |  |  |  | .13 | .02 | .00 | -.03 | .01 | .01 | -.01 | .01 | .00 | -.01 | .03 | .01 | .01 | .00 | .05 | .04 | -.07 | .03 | .00 | -.02 | .01 | .05 |
| Health change Physical |  |  |  |  |  |  | .19 | -.01 | -.02 | .03 | .02 | .02 | .01 | .01 | -.02 | -.01 | .04 | .02 | .04 | .00 | -.02 | -.06 | .16 | .00 | .02 | .04 | .00 |
| Health change Mental |  |  |  |  |  |  |  | .00 | .01 | .00 | .01 | -.03 | -.03 | .00 | .02 | .02 | .00 | .01 | .03 | -.01 | -.01 | -.02 | .11 | -.13 | -.07 | -.04 | .06 |
| Risk Perception |  |  |  |  |  |  |  |  | .28 | -.03 | .05 | .07 | .05 | .11 | .02 | -.01 | .01 | .04 | .01 | -.03 | .01 | .04 | .03 | .01 | -.01 | .01 | .00 |
| Health Risk |  |  |  |  |  |  |  |  |  | -.05 | .06 | .00 | .03 | .13 | -.02 | .03 | -.02 | .00 | .04 | -.02 | .01 | .02 | -.03 | -.02 | .02 | .01 | .00 |
| Self-exempting Beliefs |  |  |  |  |  |  |  |  |  |  | .02 | -.02 | .03 | -.02 | .04 | -.03 | .04 | -.03 | .01 | .05 | -.01 | .02 | .01 | -.01 | .01 | -.01 | -.01 |
| Economic Consequences |  |  |  |  |  |  |  |  |  |  |  | .06 | .06 | .04 | .13 | -.04 | .00 | .02 | -.03 | .02 | .02 | -.01 | .01 | -.01 | .01 | .02 | -.01 |
| Involvement |  |  |  |  |  |  |  |  |  |  |  |  | .16 | .12 | .02 | .04 | .06 | .10 | -.03 | .00 | .09 | -.01 | -.01 | .03 | .02 | -.01 | -.02 |
| Negative Affect |  |  |  |  |  |  |  |  |  |  |  |  |  | .15 | .19 | -.04 | -.01 | -.07 | -.05 | -.01 | .01 | .02 | .02 | .09 | .09 | .04 | -.04 |
| Worries Virus |  |  |  |  |  |  |  |  |  |  |  |  |  |  | .24 | .02 | .04 | .08 | -.03 | -.04 | .05 | .02 | -.01 | .01 | .02 | -.01 | -.01 |
| Worries Measures |  |  |  |  |  |  |  |  |  |  |  |  |  |  |  | .00 | .02 | -.01 | -.04 | .00 | .00 | .04 | -.04 | .09 | -.01 | .09 | -.03 |
| Trust |  |  |  |  |  |  |  |  |  |  |  |  |  |  |  |  | .09 | .26 | -.02 | .04 | .03 | -.01 | .01 | -.02 | -.01 | -.01 | .04 |
| Social norm |  |  |  |  |  |  |  |  |  |  |  |  |  |  |  |  |  | .12 | -.02 | .01 | .13 | .02 | .00 | .01 | .01 | .02 | -.02 |
| Measures Support |  |  |  |  |  |  |  |  |  |  |  |  |  |  |  |  |  |  | .20 | .00 | .22 | .02 | .00 | .00 | .01 | -.01 | .01 |
| Measures Ease |  |  |  |  |  |  |  |  |  |  |  |  |  |  |  |  |  |  |  | .11 | .01 | .03 | .01 | -.02 | -.03 | -.02 | .01 |
| Control Infection |  |  |  |  |  |  |  |  |  |  |  |  |  |  |  |  |  |  |  |  | .05 | .01 | .01 | .02 | -.01 | -.02 | .03 |
| Compliance |  |  |  |  |  |  |  |  |  |  |  |  |  |  |  |  |  |  |  |  |  | .00 | .02 | .03 | .01 | .02 | .03 |
| Somatic Complaints |  |  |  |  |  |  |  |  |  |  |  |  |  |  |  |  |  |  |  |  |  |  | -.03 | .07 | .16 | -.02 | -.02 |
| Healthy Lifestyle |  |  |  |  |  |  |  |  |  |  |  |  |  |  |  |  |  |  |  |  |  |  |  | -.01 | -.03 | .01 | .02 |
| Depressive Complaints |  |  |  |  |  |  |  |  |  |  |  |  |  |  |  |  |  |  |  |  |  |  |  |  | .34 | .19 | -.10 |
| Anxiety Complaints |  |  |  |  |  |  |  |  |  |  |  |  |  |  |  |  |  |  |  |  |  |  |  |  |  | .08 | -.09 |
| Loneliness |  |  |  |  |  |  |  |  |  |  |  |  |  |  |  |  |  |  |  |  |  |  |  |  |  |  | -.15 |
| Mental Wellbeing |  |  |  |  |  |  |  |  |  |  |  |  |  |  |  |  |  |  |  |  |  |  |  |  |  |  |  |

**Node strength**

|  | Temporal network | | Contemporaneous network |
| --- | --- | --- | --- |
| Node | InStrength | OutStrength | Strength |
| Perceived Knowledge | -1.23 | -0.5 | -1.19 |
| Compassion | 0.56 | -0.55 | -0.33 |
| Self-efficacy | -0.96 | -0.79 | -1.25 |
| Vaccine Intention | -0.32 | 0.42 | -1.07 |
| Health General | -0.95 | -1.04 | -1 |
| Health change Physical | -0.32 | -0.07 | -0.04 |
| Health change Mental | -0.66 | -0.88 | -0.07 |
| Risk Perception | -0.14 | -0.75 | 0.04 |
| Health Risk | -0.29 | -0.52 | -0.09 |
| Self-exempting Beliefs | -1.05 | -0.47 | -1.38 |
| Economic Consequences | -0.1 | -0.78 | -0.85 |
| Involvement | 1.87 | 2.48 | 1.36 |
| Negative Affect | 1.69 | 0.08 | 1.96 |
| Worries Virus | 1.72 | 1.05 | 1.56 |
| Worries Measures | 0.97 | -0.22 | 1.01 |
| Trust | -0.49 | -0.78 | 0.33 |
| Social norm | 1.21 | 1.05 | -0.35 |
| Measures Support | 0.97 | 2.26 | 2.16 |
| Measures Ease | -0.89 | -0.61 | -0.27 |
| Control Infection | -1.23 | -0.97 | -0.97 |
| Compliance | 1.75 | 1.79 | 0.32 |
| Somatic Complaints | -0.51 | -0.46 | -0.6 |
| Healthy Lifestyle | -1.19 | -0.6 | -0.9 |
| Depressive Complaints | 0.47 | 0.66 | 1.14 |
| Anxiety Complaints | -0.15 | 0.96 | 0.7 |
| Loneliness | -0.21 | -0.02 | -0.1 |
| Mental Wellbeing | -0.5 | -0.76 | -0.15 |

## 2.5 Edge accuracy of COVID-19 broad attitude networks

The figures below present the edge bootstrap confidence intervals, which are an indication of edge accuracy. Generally speaking, the confidence intervals of the edge weights were not wide, indicating stable (reliable) edges.

**Edge accuracy temporal COVID-19 network**


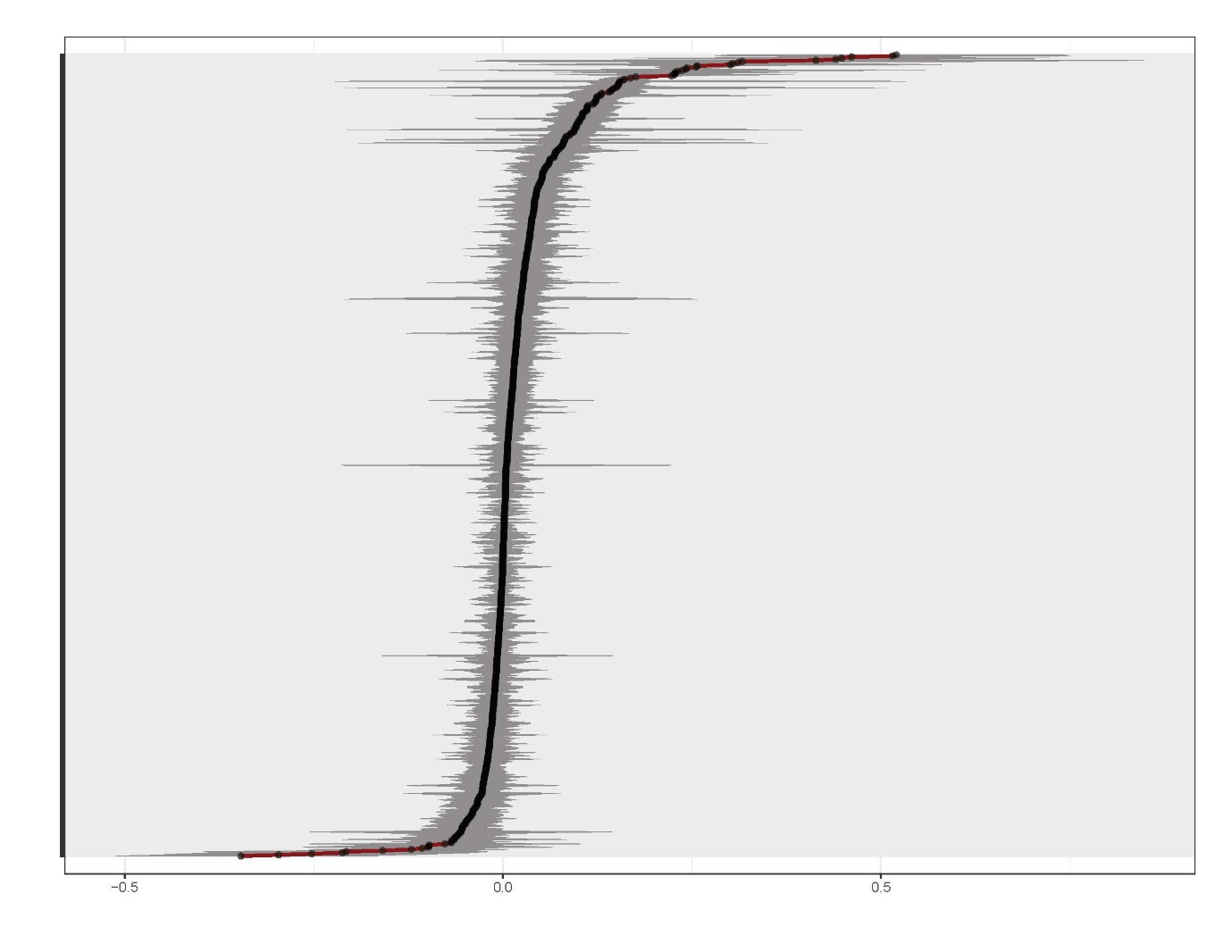


Please note that the edges in the temporal COVID-19 network presented in the manuscript represent partial directed correlations (PDC), which are *standardized* beta values. The figure presented here contains the beta values and their confidence intervals of the edges of the temporal network.

**Edge accuracy contemporaneous COVID-19 network**


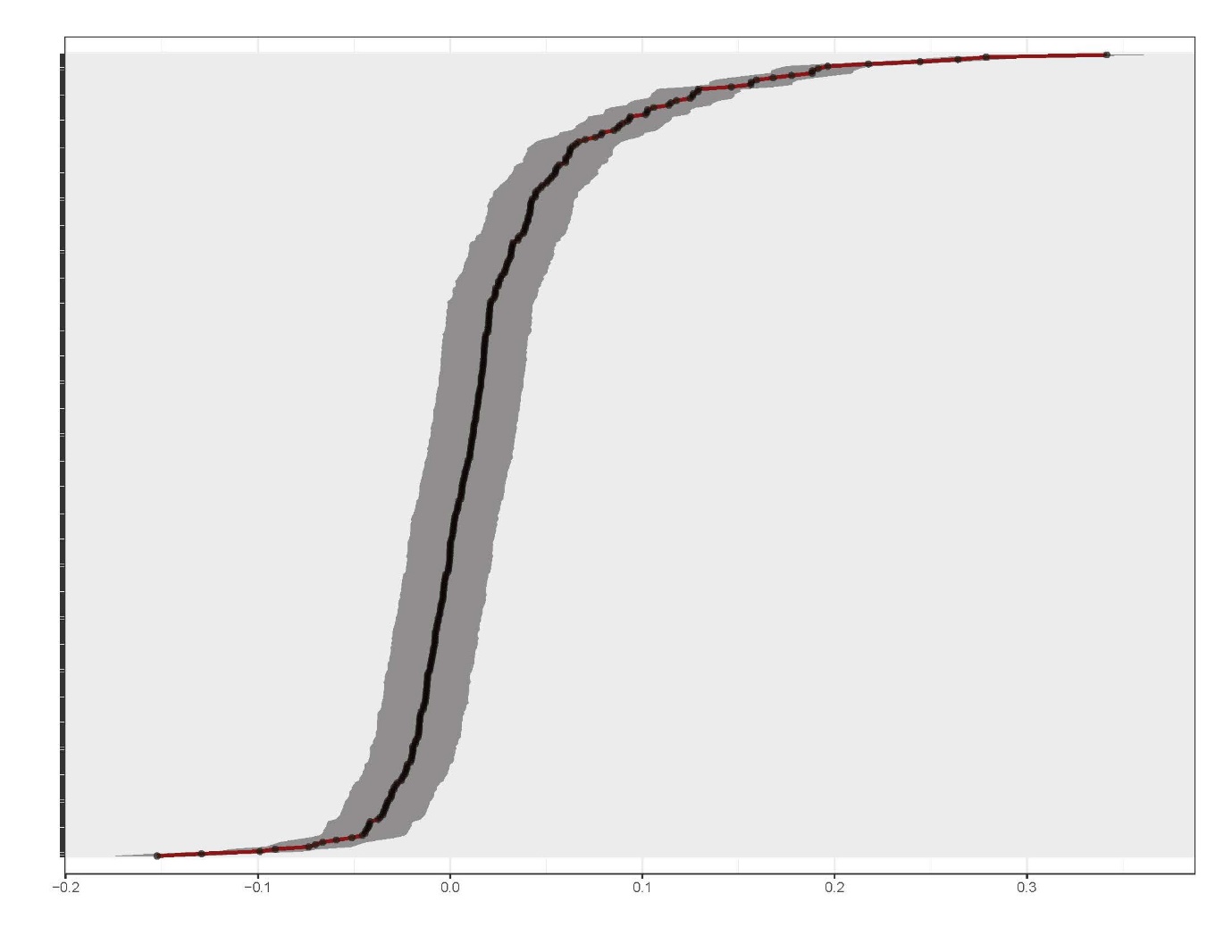


## 2.6 Coherence within-person COVID-19 broad attitude networks

Plot of relation between edges of the temporal and contemporaneous COVID-19 networks (*r* = 0.47, *z* = 0.51). A Fisher’s *r*-to-*z* transformation was included to approximate normal distribution.


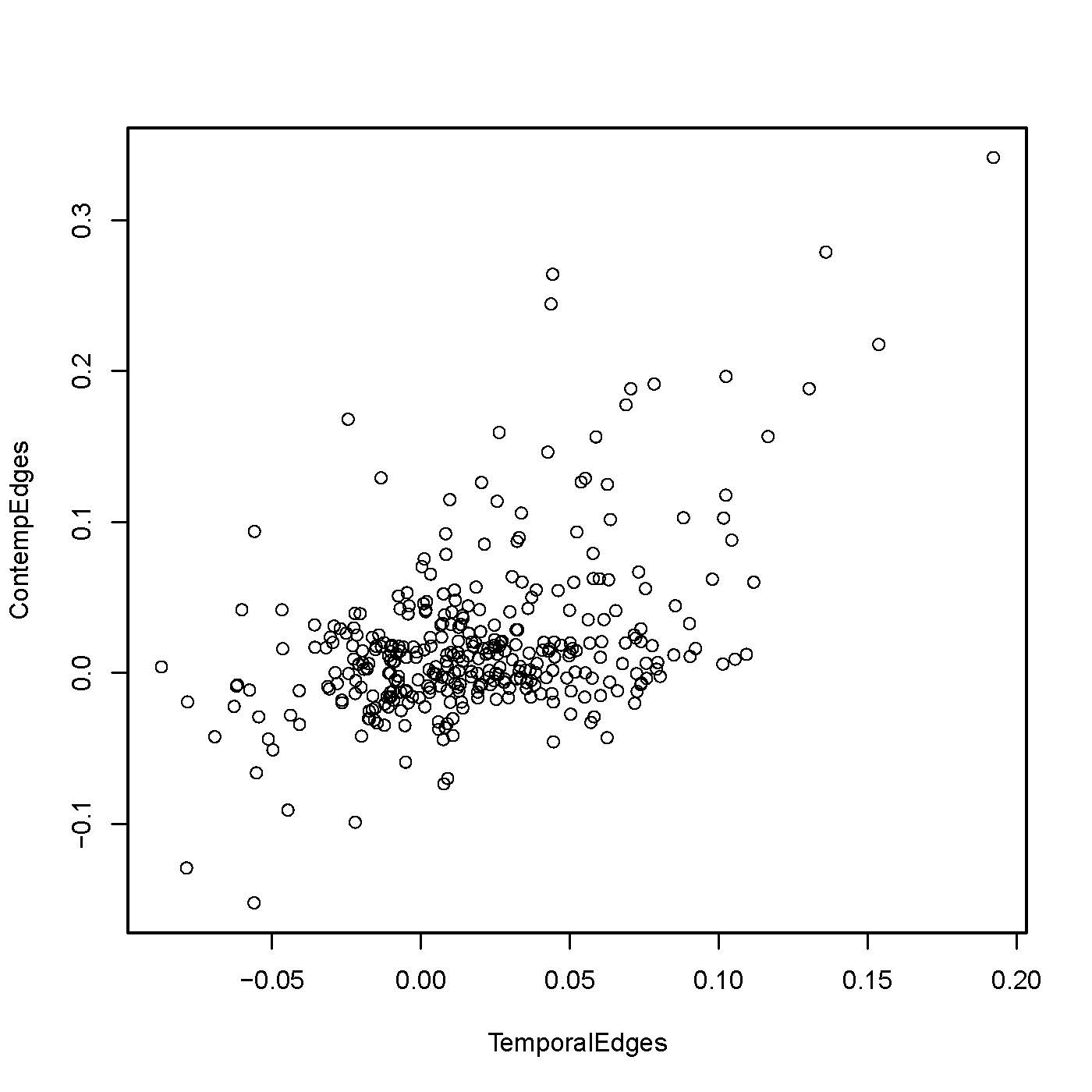


# Timeline COVID-19 in the Netherlands

| Date | NL/international | Theme (statistics/safety measures/news/research) | Description of the event | Source |
| --- | --- | --- | --- | --- |
| 31-12-2019 | International | Statistics | WHO is informed of cases of pneumonia of unknown cause detected in the city of Wuhan, in the Hubei Province, China. | WHO: http://www.euro.who.int/en/health-topics/health-emergencies/international-health-regulations/news/news/2020/2/novel-coronavirus-emerges-in-china |
| 10-1-2020 | International | Statistics | The WHO reports that a novel coronavirus has emerged in China. After the first reports of pneumonia came in China came in, it has been confirmed by Chinese authorities that this is caused by a novel coronavirus. | WHO: http://www.euro.who.int/en/health-topics/health-emergencies/international-health-regulations/news/news/2020/2/novel-coronavirus-emerges-in-china |
| 30-1-2020 | International | Safety measures | The WHO declares the coronavirus outbreak as a global emergency, demanding temporary safety measures to be made in all countries. Countries have to prove their healthcare safety measures are based on sound scientific reasoning. According to the WHO, declaring an international emergency helps strengthen global coordination, transparency and increases support for countries with less developed healthcare systems. | WHO: http://www.euro.who.int/en/health-topics/health-emergencies/international-health-regulations/news/news/2020/2/2019-ncov-outbreak-is-an-emergency-of-international-concern |
| 31-1-2020 | International | Statistics | First case of coronavirus confirmed in Spain. | CNN: https://edition.cnn.com/asia/live-news/coronavirus-outbreak-02-01-20-intl-hnk/h_afcf3a4665521aab11c721c8cc80dd03 ; El Pais: https://elpais.com/sociedad/2020/01/31/actualidad/1580509404_469734.html |
| 2-2-2020 | NL | News | Flight with over 15 Dutch people returns from Wuhan after waiting several days to be able to return. | Volkskrant: https://www.volkskrant.nl/nieuws-achtergrond/verslag-van-de-evacuatie-uit-wuhan-bij-de-douane-loop-je-een-rampenfilm-binnen~bb1dba1b/ ; Telegraaf: https://www.telegraaf.nl/nieuws/455667699/nederlanders-uit-coronastad-wuhan-moeten-eerst-tot-rust-komen |
| 7-2-2020 | International | News | Dr Wenliang, a Chinese doctor who tried to warn people about the possible spread of the virus, died from the coronavirus. | BBC: https://www.bbc.com/news/world-asia-china-51403795 ; The Guardian: https://www.theguardian.com/global-development/2020/feb/07/coronavirus-chinese-rage-death-whistleblower-doctor-li-wenliang |
| 11-2-2020 | International | News | The WHO announces official names for the novel coronavirus disease (COVID-19) and the virus that causes it (SARS-CoV-2). | WHO: https://www.who.int/emergencies/diseases/novel-coronavirus-2019/technical-guidance/naming-the-coronavirus-disease-(covid-2019)-and-the-virus-that-causes-it |
| 23-2-2020 | NL | Safety measures | After the virus starts to spread in other European countries like Italy, the Dutch government is not yet implementing extra safety measures in the Netherlands. Meanwhile, the Dutch Carnival has started in most southern parts of the Netherlands, with large crowds of people coming together to celebrate. | Volkskrant: https://www.volkskrant.nl/nieuws-achtergrond/uitbraak-coronavirus-in-noord-italie-geen-extra-maatregelen-in-nederland~b6518425/ |
| 27-2-2020 | NL | Statistics | A first case of the new coronavirus has been confirmed by the National Institute for Public Health and Environment (RIVM). The patient is a 56 year old man living in Brabant, a southern province of the Netherlands. He most likely contracted the virus on a trip to the Lombardy region in Italy. | RIVM: https://www.rivm.nl/nieuws/patient-met-nieuw-coronavirus-in-nederland ; Volkskrant: https://www.volkskrant.nl/nieuws-achtergrond/burgemeester-tilburg-coronapatient-tilburg-was-niet-besmettelijk-tijdens-carnaval~b5510762/; Telegraaf: https://www.telegraaf.nl/nieuws/214089776/rivm-eerste-coronageval-in-nederland |
| 28-2-2020 | NL | Statistics | A second case of the coronavirus has been confirmed by the RIVM. The patient is a woman living in Amsterdam, who returned from a trip to the Lombardy region in Italy | RIVM: https://www.rivm.nl/nieuws/tweede-patient-in-nederland-met-covid-19 ; WHO: https://covid19.who.int/region/euro/country/nl |
| 4-3-2020 | NL | Safety measures | The RIVM advises for "only necessary travels" to all of Northern Italy. Many big companies in the Netherlands start to take measures against the virus, even though this is not yet required by authorities. | RIVM: https://www.rivm.nl/coronavirus-covid-19/actueel ; Volkskrant: https://www.volkskrant.nl/nieuws-achtergrond/veel-thuiswerken-en-niet-meer-reizen-internationale-bedrijven-nemen-maatregelen-tegen-corona~b773241c/ ; |
| 4-3-2020 | International | Safety measures | Italy announces it is closing down schools and universities nationwide until mid-march. | The Guardian https://www.theguardian.com/world/2020/mar/04/italy-orders-closure-of-schools-and-universities-due-to-coronavirus; Independent: https://www.independent.co.uk/news/world/europe/coronavirus-italy-school-university-closed-outbreak-death-toll-health-latest-a9374801.html |
| 5-3-2020 | NL | Statistics | The number of positive tested patients in the Netherlands reportedly grew with 41, more than doubling the total amount to 82 patients. According to Bruno Bruins, minister of medical care, this number is due to a catch-up in conducted tests. | WHO: https://covid19.who.int/region/euro/country/nl ; RIVM: https://www.rivm.nl/coronavirus-covid-19/actueel ; AD: https://www.ad.nl/binnenland/aantal-coronapatienten-in-nederland-ruim-verdubbeld-naar-82~a886fdb8/?referrer=https://en.wikipedia.org/ |
| 6-3-2020 | NL | Statistics | A first patient with COVID-19 dies in the Netherlands. The patient was an 86 year old man who lived in Hoeksche Waard, Zuid-Holland. Total cases grows to: 128 | WHO: https://covid19.who.int/region/euro/country/nl ; RIVM: https://www.rivm.nl/nieuws/patient-met-nieuw-coronavirus-overleden ; Parool: https://www.parool.nl/nederland/eerste-dode-door-coronavirus-in-nederland~b8d72ed6/?referer=https%3A%2F%2Fnl.wikipedia.org%2F |
| 6-3-2020 | NL | Safety measures | RIVM advises people in Noord-Brabant to limit their social contacts in case of colds, coughs and fevers. | RIVM: https://www.rivm.nl/nieuws/covid-19-nieuwe-aanwijzing-voor-inwoners-noord-brabant |
| 9-3-2020 | International | Safety measures | Italy's prime minister Guiseppe Conte announces a nationwide forced quarantine on national television. Only people with a valid work or family reason are allowed to travel. All social events are cancelled. | BBC: https://www.bbc.com/news/world-europe-51810673 ; The Wall Street Journal: https://www.wsj.com/articles/italy-bolsters-quarantine-checks-after-initial-lockdown-confusion-11583756737 |
| 9-3-2020 | NL | Safety measures | Prime minister Mark Rutte holds a nationally televised press conference with Jaap van Dissel, RIVM director of disease control, to inform people on the coronavirus. In this press conference, he advises people to stop shaking hands and to work from home if you live in Noord-Brabant. Earlier that day, the government also advised to regularly wash your hands, cough or sneeze in your elbow and to only make use of paper towels. After the press conference ended, the PM mistakenly shook hands with Jaap van Dissel. | NOS: https://nos.nl/video/2326490-rutte-stop-met-handen-schudden-en-werk-thuis.html ; Parool: https://www.parool.nl/nederland/rutte-reikt-rivm-de-hand-na-oproep-om-geen-handen-meer-te-schudden~ba6d39c9/ ; RIVM: https://www.rivm.nl/coronavirus-covid-19/actueel |
| 11-3-2020 | International | News | The WHO declares that COVID-19 can be characterized as a pandemic. This does not change the WHO's assessment of the threat posed by the virus, neither does it change what the WHO's actions are towards solving the situation. According to the WHO, the characterization as a pandemic serves to warn countries and to call for countries to 'take urgent and aggressive action'. | WHO: https://www.who.int/dg/speeches/detail/who-director-general-s-opening-remarks-at-the-media-briefing-on-covid-19---11-march-2020 |
| 12-3-2020 | NL | Statistics | The total number of patients that have tested positive has grown with 111, bringing the total number of cases to 614. The total number of patients that have died is 5. | WHO: https://covid19.who.int/region/euro/country/nl (retrieved on 29-06-2020) |
| 12-3-2020 | NL | Safety measures | The RIVM announces that the safety measures implemented in the province of Noord-Brabant now apply to the whole of the Netherlands. Events with over 100 people are banned at least until 31st of March, schools remain open. | RIVM: https://www.rivm.nl/nieuws/uitbreiding-maatregelen-coronavirus ; https://www.rijksoverheid.nl/onderwerpen/coronavirus-covid-19/nieuws/2020/03/12/nieuwe-maatregelen-tegen-verspreiding-coronavirus-in-nederland ; |
| 14-3-2020 | International | Safety measures | The Spanish government has formally declared a state of emergency over the coronavirus, placing the country in lockdown for the next weeks. | The Guardian: https://www.theguardian.com/world/2020/mar/14/spain-government-set-to-order-nationwide-coronavirus-lockdown ; Businessinsider: https://www.businessinsider.com/coronavirus-spain-nationalises-private-hospitals-emergency-covid-19-lockdown-2020-3?international=true&r=US&IR=T |
| 15-3-2020 | NL | Safety measures | In a press conference, minister Bruins and minister of education Arie Slob announces that schools are closed and teachers have to prepare remote teaching for the children at home. Restaurants, bars, sports clubs and more establishments have to close until April 8. People are asked to keep a distance of 1,5 meters to each other where possible. | Government: https://www.rijksoverheid.nl/actueel/nieuws/2020/03/15/aanvullende-maatregelen-onderwijs-horeca-sport |
| 16-3-2020 | NL | Statistics | The total number of patients that have tested positive has grown with 278 and 4 patients have died. This brings the total number of cases to 1,413 and the number of patients that died to 24. | WHO: https://covid19.who.int/region/euro/country/nl (retrieved on 29-06-2020) |
| 16-3-2020 | NL | Safety measures | PM Rutte addresses the nation about the coronavirus. In his speech, he announced that the government will not resort to a full lockdown, but instead to opt for a method of controlled spread of the virus. This method aims to build group immunity, but Rutte also stated that a large proportion of the Dutch population will be infected because of this. | NOS: https://www.youtube.com/watch?v=D3QPmphUK_M ; https://nos.nl/artikel/2327309-de-komende-tijd-zal-een-groot-deel-van-de-bevolking-met-het-virus-besmet-raken.html |
| 16-3-2020 | NL | News | There were positive reactions to the sign language interpreter, because of the gestures she made for the word 'hamsteren' (hoarding). | RTL: https://www.rtlnieuws.nl/nieuws/nederland/artikel/5057841/gebarentolk-irma-sluis-hamsteren-persconferentie-coronavirus ; Volkskrant: https://www.volkskrant.nl/nieuws-achtergrond/voor-het-eerst-een-doventolk-bij-een-belangrijke-persconferentie-het-gebaar-voor-hamsteren-moest-ik-wel-even-opzoeken~be0bdbcc/ |
| 17-3-2020 | NL | News | In the evening of the 17th, many Dutch people applauded the people with necessary jobs, like care workers, garbage collectors and others. | Parool: https://www.parool.nl/nederland/oproep-klap-je-handen-stuk-voor-iedereen-die-nederland-door-coronacrisis-slaat~b874fe2a/?referer=https%3A%2F%2Fwww.google.nl%2F ; https://www.nrc.nl/nieuws/2020/03/17/applaus-voor-zorgmedewerkers-a3994068 |
| 17-3-2020 | NL | News | People have started hoarding certain products, like toilet paper and pasta, from supermarkets around the country. | Trouw: https://www.trouw.nl/economie/nederland-blijft-wc-papier-blikgroente-en-zeep-hamsteren~bce1b5ff/ ; NRC: https://www.nrc.nl/nieuws/2020/03/30/na-persconferenties-van-rutte-begint-het-hamsteren-zien-ze-bij-jumbo-a3995329 ; FD: https://fd.nl/ondernemen/1338423/nederlandse-supermarkten-nemen-maatregelen-tegen-hamsteren-en-besmetting |
| 17-3-2020 | NL | News | In a newsletter to the public, the cabinet announces that they will take exceptional economic measures to accommodate the jobs and incomes of self-employed workers, entrepreneurs and large companies. | Government: https://www.rijksoverheid.nl/actueel/nieuws/2020/03/17/coronavirus-kabinet-neemt-pakket-nieuwe-maatregelen-voor-banen-en-economie |
| 19-3-2020 | NL | Statistics | The total number of patients that have tested positive has grown with 409 and the deaths have grown with 18. This brings the total number of cases to 2,460 and the number of people that died to 76. | WHO: https://covid19.who.int/region/euro/country/nl (retrieved on 29-06-2020) |
| 19-3-2020 | NL | News | Bruno Bruins resigns as minister of medical care due to the workload being too taxing. The minister collapsed the previous night during a debate on the coronavirus. Minister Hugo de Jonge takes over his coronavirus-related tasks. | RTL: https://www.rtlnieuws.nl/nieuws/politiek/artikel/5062871/bruins-minister-ontslag ; AD: https://www.ad.nl/politiek/plichtsgetrouwe-minister-bruins-stapt-op-coronacrisis-wordt-hem-nu-toch-te-machtig-br~a324a364/ |
| 20-3-2020 | NL | News | King of the Netherlands Willem Alexander addressed the nation on national television, telling people that they cannot stop the coronavirus, but they can stop the 'loneliness-virus'. | NOS: https://nos.nl/artikel/2327794-koning-coronavirus-kunnen-we-niet-stoppen-eenzaamheidsvirus-wel.html |
| 22-3-2020 | NL | Safety measures | The Dutch government has sent an 'NL-alert' text message to a large proportion of Dutch citizens, telling them to keep 1.5m distance and remain at home if you are sick. | Government: https://www.rijksoverheid.nl/actueel/nieuws/2020/03/22/nl-alert-met-instructies-tegen-coronavirus |
| 23-3-2020 | NL | Statistics | The total number of patients that have tested positive has grown with 573 and the deaths have grown with 43. This brings the total number of cases to 4,204 and the number of people that died to 179. | WHO: https://covid19.who.int/region/euro/country/nl (retrieved on 29-06-2020) |
| 23-3-2020 | NL | Safety measures | In a nationally televised press conference, PM Rutte introduces new safety measures, calling it an 'intelligent lockdown'. This means that people are still allowed to go outside as long as they keep 1.5 meters distance. Other new safety measures include that all events are cancelled until June 1st and people are not allowed to meet more than 3 other people outside or in their homes. The government also gave more power to local authorities to hand out fines to people that do not follow the instructions (up to €390 for individuals). | NOS: https://nos.nl/video/2328097-premier-rutte-dit-is-een-intelligente-lockdown.html ; Parool: https://www.parool.nl/nederland/dit-was-het-belangrijkste-coronanieuws-van-23-maart~bf16457b/ |
| 24-3-2020 | NL | Safety measures | In a letter to the house of representatives, the minister of education announces all central final exams in May are cancelled. This is the first time since 1945 that this has happened in the Netherlands. | Government: https://www.tweedekamer.nl/kamerstukken/brieven_regering/detail?id=2020Z05514&did=2020D11480 |
| 25-3-2020 | NL | Safety measures | The government made guidelines for responsible shopping to instruct stores on how to keep their businesses running in a way that is safe for both employees and customers. | Government: https://www.rijksoverheid.nl/actueel/nieuws/2020/03/25/nieuwe-regels-voor-verantwoord-winkelen |
| 26-3-2020 | NL | Statistics | The total number of patients that have tested positive has grown with 852 and the deaths have grown with 80. This brings the total number of cases to 6,412 and the number of people that died to 365. | WHO: https://covid19.who.int/region/euro/country/nl (retrieved on 29-06-2020) |
| 26-3-2020 | NL | News | The Government starts a new campaign that aims to offer people a clear and calming message. The campaign is called 'Alleen samen' (Only together) and is displayed on various media platforms like television, social media, websites. | RTL: https://www.rtlnieuws.nl/editienl/artikel/5070981/campagne-slogan-rijksoverheid-coronacrisis-alleen-samen ; Marketing tribune: https://www.marketingtribune.nl/bureaus/nieuws/2020/03/alleen-samen/index.xml |
| 26-3-2020 | NL | News | The government's central planning office (CPB) made four scenarios for the economic impact of the coronavirus pandemic and states that a recession is inevitable. | CPB: https://www.cpb.nl/scenarios-coronacrisis |
| 31-3-2020 | NL | Statistics | The total number of patients that have tested positive has grown with 884 and the deaths have grown with 93. This brings the total number of cases to 11,750 and the number of people that died to 864. The RIVM reported that the number of reported patients is still growing, but growing less hard. | WHO: https://covid19.who.int/region/euro/country/nl (retrieved on 29-06-2020) |
| 31-3-2020 | NL | Safety measures | In a press conference with Hugo de Jonge, minister of health, Mark Rutte announces that all safety measures initiated on 15 March will be extended until at least 28 April. | Government: https://www.youtube.com/watch?v=-m5gkmETnV0 ; NU.nl: https://www.nu.nl/coronavirus/6041538/noodmaatregelen-verlengd-scholen-en-horeca-blijven-dicht-tm-28-april.html |
| 2-4-2020 | NL | Statistics | The total number of patients that have tested positive has grown with 1,019 and the deaths have grown with 134. This brings the total number of cases to 13,614 and the number of people that died to 1,173. | WHO: https://covid19.who.int/region/euro/country/nl (retrieved on 29-06-2020) |
| 7-4-2020 | NL | Safety measures | In a nationally televised press conference, PM Rutte says that the measures could possibly be loosened a bit after 28 April, however getting back to 'normal' will take considerable time. | Volkskrant: https://www.volkskrant.nl/nieuws-achtergrond/rutte-terug-naar-normaal-gaat-lang-duren~bd06f4f5/ ; Parool: https://www.parool.nl/wereld/dit-was-het-belangrijkste-coronanieuws-van-dinsdag-7-april~bfe1a094/ |
| 9-4-2020 | NL | Statistics | The total number of patients that have tested positive has grown with 969 and the deaths have grown with 147. This brings the total number of cases to 20,549 and the number of people that died to 2,248. | WHO: https://covid19.who.int/region/euro/country/nl (retrieved on 29-06-2020) |
| 11-4-2020 | NL | News | The Ministry of Health, Welfare and Sport invites companies and experts to contribute ideas on the development and deployment of apps in the fight against the coronavirus. The apps can be used for tracing infections and reporting health problems. | Government: https://www.rijksoverheid.nl/actueel/nieuws/2020/03/17/coronavirus-kabinet-neemt-pakket-nieuwe-maatregelen-voor-banen-en-economie |
| 15-4-2020 | NL | News | PM Rutte said in a press conference that the cabinet is considering to loosen safety measures in the near future. This can only happen if the following conditions are met: the pressure on healthcare must be reduced, the elderly must be adequately protected and there must be 'insight into the spread of the virus'. | AD: https://www.ad.nl/politiek/rutte-het-loket-gaat-zeker-niet-in-een-keer-open~ada8a36a/?referrer=https://www.google.com/ ; Parool: https://www.parool.nl/nederland/rutte-het-loket-gaat-zeker-niet-in-een-keer-open~bda8a36a/ |
| 16-4-2020 | NL | Statistics | The total number of patients that have tested positive has grown with 734 and the deaths have grown with 189. This brings the total number of cases to 28,153 and the number of people that died to 3,134. | WHO: https://covid19.who.int/region/euro/country/nl (retrieved on 29-06-2020) |
| 21-4-2020 | NL | Statistics | The total number of patients that have tested positive has grown with 750 and the deaths have grown with 67. This brings the total number of cases to 33,405 and the number of people that died to 3,751. | WHO: https://covid19.who.int/region/euro/country/nl (retrieved on 29-06-2020) |
| 21-4-2020 | NL | Safety measures | In a press conference, PM Rutte announces that schools for children up to 12 can be partially reopened. Children up to 12 are allowed to play organized sports under supervision and children up to 18 can play team sports when keeping a distance. The other initial measures remain until 20 May. All events are cancelled until 1st of September. | Government: https://www.rijksoverheid.nl/actueel/nieuws/2020/04/21/maatregelen-corona-verlengd |
| **23-4-2020** | **NL** | **Research** | **Start data collection wave 1** |  |
| 24-4-2020 | NL | News | The ministry of Finance announces that the national budget deficit will be 92 billion euros or 11.8 percent. This is the highest deficit since the second world war. | AD: https://www.ad.nl/politiek/coronacrisis-is-dreun-voor-de-schatkist-grootste-tekort-sinds-tweede-wereldoorlog~a8102f15/?referrer=https://www.google.com/ ; Telegraaf: https://www.telegraaf.nl/nieuws/308026519/duizelingwekkend-begrotingstekort-92-miljard |
| 27-4-2020 | NL | News | The government is working on an emergency law to increase the legal grounds of the corona measures. | NOS: https://nos.nl/artikel/2331870-kabinet-werkt-aan-spoedwet-nu-noodmaatregelen-langer-gaan-duren.html; NRC https://www.nrc.nl/nieuws/2020/04/27/spoedwet-voor-noodmaatregelen-a3997955 |
| 28-4-2020 | NL | Statistics | The total number of patients that have tested positive has grown with 400 and the deaths have grown with 43. This brings the total number of cases to 38,245 and the number of people that died to 4,518. | WHO: https://covid19.who.int/region/euro/country/nl (retrieved on 29-06-2020) |
| 28-4-2020 | NL | News | More than 100 people demonstrate in The Hague against the corona measures, more specifically the lockdown. | NOS: https://nos.nl/video/2332030-demonstratie-tegen-lockdownmaatregelen-van-het-nederlandse-kabinet.html; Nu.nl: https://www.nu.nl/coronavirus/6047702/ruim-honderd-mensen-bij-demonstratie-tegen-coronamaatregelen-in-den-haag.html?redirect=1 |
| 5-5-2020 | NL | Statistics | The total number of patients that have tested positive has grown with 199 and the deaths have grown with 26. This brings the total number of cases to 40,770 and the number of people that died to 5,082. | WHO: https://covid19.who.int/region/euro/country/nl (retrieved on 29-06-2020) |
| 5-5-2020 | NL | News | Several demonstrations are organized against the corona measures. An unauthorized demonstration with (estimated) 300 people in The Hague led to eighty arrests. The demonstration was not reported to the municipality. | NOS: https://nos.nl/artikel/2332855-tachtig-arrestaties-in-den-haag-na-beeindigen-anti-lockdowndemonstratie.html; AD: https://www.ad.nl/binnenland/tachtig-anti-lockdownactivisten-aangehouden-bij-demonstratie-in-den-haag~aa966a30/ |
| **5-5-2020** | **NL** | **Research** | **End data collection wave 1** |  |
| 6-5-2020 | NL | Safety measures | In a press conference, PM Rutte announces a plan to loosen the safety measures step by step. The new message for the people is: "Stay home if you experience symptoms" and "avoid crowded areas". | Government: https://www.rijksoverheid.nl/onderwerpen/coronavirus-covid-19/vraag-en-antwoord/de-persconferentie-van-6-mei-in-eenvoudige-taal ; https://www.rijksoverheid.nl/actueel/nieuws/2020/05/06/vermijd-drukte-houd-15-meter-afstand |
| 11-5-2020 | NL | Safety measures | The first cautious step are being taken towards loosening the visiting arrangements for nursing homes. In certain nursing homes, they will allow one visitor per resident as a trial. | Government: https://www.rijksoverheid.nl/actueel/nieuws/2020/05/11/deelnemende-instellingen-versoepeling-bezoekregeling-verpleeghuizen-bekend |
| 11-5-2020 | NL | Safety measures | Hairdressers and beauty salons are allowed to reopen, but only if they can guarantee the safety measures are complied with. | RTL: https://www.rtlnieuws.nl/economie/business/artikel/5118746/kappers-open-corona-hygiene-toeslag-maatregelen-klanten ; Nu.nl: https://www.nu.nl/economie/6050458/kappers-weer-open-met-ruimere-openingstijden-en-hogere-prijzen.html |
| 12-5-2020 | NL | Statistics | The total number of patients that have tested positive has grown with 161 and the deaths have grown with 16. This brings the total number of cases to 42,788 and the number of people that died to 5,456. | WHO: https://covid19.who.int/region/euro/country/nl (retrieved on 29-06-2020) |
| **13-5-2020** | **NL** | **Research** | **Start data collection wave 2** |  |
| 14-5-2020 | NL | News | > 10,000 corona fines have been handed out (see 23-03-2020), resistance increases | NOS: https://nos.nl/artikel/2333914-fouten-bij-uitschrijven-coronaboetes-deel-kan-niet-geind-worden.html;v Volkskrant: https://www.volkskrant.nl/nieuws-achtergrond/groeiend-verzet-tegen-coronaboetes-al-ruim-10-duizend-bekeuringen-uitgedeeld~bc97c8efa/ |
| 15-5-2020 | NL | News | First cases of infected pets in NL (3 cats and one dog). | NOS: https://nos.nl/artikel/2334019-coronavirus-vastgesteld-bij-drie-katten-en-een-hond.html; Volkskrant: https://www.volkskrant.nl/nieuws-achtergrond/voor-het-eerst-in-nederland-huisdieren-besmet-met-corona-virus-aangetroffen-bij-katten-en-een-hond~b4d81036/ |
| **18-5-2020** | **NL** | **Research** | **End data collection wave 2** |  |
| 19-5-2020 | NL | Safety measures | The government announces that safety measures still apply, but certain measures can be made more flexible. Groups of people are allowed to meet outside, when keeping 1,5 m distance. Cafés/restaurants and secondary schools are partially reopened on June 1st as planned. Primary schools will be completely reopened on June 8. Not wearing a face mask in public transport will result in a €95 fine. | Government: https://www.rijksoverheid.nl/actueel/nieuws/2020/05/19/corona-aanpak-de-volgende-stap |
| 19-5-2020 | NL | Statistics | The total number of patients that have tested positive has grown with 146 and the deaths have grown with 14. This brings the total number of cases to 44,141 and the number of people that died to 5,694. | WHO: https://covid19.who.int/region/euro/country/nl (retrieved on 11-08-2020) |
| 21-5-2020 | NL | News | It is getting busier at outdoor locations (e.g., parks and beaches). Park gets evacuated due to it being overcrowded. | NOS: https://nos.nl/artikel/2334600-drukte-op-hemelvaartsdag-parken-ontruimd.html; NU.nl: https://www.nu.nl/den-haag/6052846/zomerse-drukte-op-strand-bij-scheveningen-op-hemelvaartsdag.html |
| 26-5-2020 | NL | Statistics | The total number of patients that have tested positive has grown with 209 and the deaths have grown with 8. This brings the total number of cases to 45,445 and the number of people that died to 5,830. | WHO: https://covid19.who.int/region/euro/country/nl (retrieved on 11-08-2020) |
| **27-5-2020** | **NL** | **Research** | **Start data collection wave 3** |  |
| 1-6-2020 | NL | Safety measures | From the 1st of June, every Dutch citizen with symptoms related to the coronavirus can get tested (free of charge). | Government: https://www.rijksoverheid.nl/actueel/nieuws/2020/05/27/vanaf-1-juni-testen-mogelijk-voor-iedereen-met-milde-klachten ; RIVM: https://www.rivm.nl/coronavirus-covid-19/testen |
| 1-6-2020 | NL | Safety measures | Bars, restaurants, museums and movie theaters partly reopen at 12:00 pm June 1st. | RTL: https://www.rtlnieuws.nl/nieuws/nederland/artikel/5139421/livestream-opening-terrassen-1-juni-juno-horeca ; Nu.nl: https://www.nu.nl/coronavirus/6055117/horeca-eindelijk-weer-open-was-wel-toe-aan-wat-anders-dan-een-tosti.html |
| 1-6-2020 | NL | News | A Black Lives Matter demonstration on the Dam square in Amsterdam has an attendance of 5,000 people. People criticized the local governments for not intervening, because this was a violation of safety measures. | Volkskrant: https://www.volkskrant.nl/nieuws-achtergrond/kritiek-op-niet-ingrijpen-van-burgemeester-bij-grote-black-lives-matter-betoging-in-amsterdam~b34ee186/ ; Telegraaf: https://www.telegraaf.nl/nieuws/1397493631/bomvolle-dam-in-amsterdam-voor-protest-tegen-amerikaans-geweld |
| **2-6-2020** | **NL** | **Research** | **End data collection wave 3** |  |
| 3-6-2020 | NL | Statistics | The total number of patients that have tested positive has grown with 102 and the deaths have grown with 5. This brings the total number of cases to 46,647 and the number of people that died to 5,967. | WHO: https://covid19.who.int/region/euro/country/nl (retrieved on 11-08-2020) |
| 3-6-2020 | NL | Safety measures | PM Mark Rutte announces that holidays inside the country are allowed again and holidays to most European countries will be allowed from June 15th | Government: https://www.rijksoverheid.nl/onderwerpen/coronavirus-covid-19/vraag-en-antwoord/persconferentie-coronavirus-zomervakantie-2020-in-eenvoudige-taal |
| 6-6-2020 | NL | News | There has been a data breach on the RIVM "Infectieradar" platform, where Dutch people can report whether they had corona-related symptoms. | NOS: https://nos.nl/artikel/2336416-lek-in-rivm-coronasite-gegevens-van-gebruikers-makkelijk-in-te-zien.html ; AD: https://www.ad.nl/tech/lek-in-rivm-coronasite-gegevens-gebruikers-makkelijk-in-te-zien~ac0a3e6b/?referrer=https://www.google.com/ |
| 9-6-2020 | NL | News | The previously announced emergency law has received much criticism. Critics say the law could violate citizens' fundamental rights. | NOS: https://nos.nl/artikel/2336696-nieuwe-coronawet-vol-verboden-krijgt-van-alle-kanten-kritiek.html; Volkskrant: https://www.volkskrant.nl/nieuws-achtergrond/grote-zorgen-over-spoedwet-corona-terug-naar-de-tijd-van-willem-i~b67b3650/ |
| 10-6-2020 | NL | Statistics | The total number of patients that have tested positive has grown with 164 and the deaths have grown with 15. This brings the total number of cases to 47,903 and the number of people that died to 6,031. | WHO: https://covid19.who.int/region/euro/country/nl (retrieved on 11-08-2020) |
| **10-6-2020** | **NL** | **Research** | **Start data collection wave 4** |  |
| 12-6-2020 | NL | News | Many hospitality entrepreneurs want to get rid of the 1.5 meter rule in cafés, bars and terraces. They say the rule does not work well and customers should be able to decide for themselves. | RTL: https://www.rtlnieuws.nl/nieuws/politiek/artikel/5150926/15-meter-horeca-onder-vuur-dit-bezopen ; Parool: https://www.parool.nl/nederland/horeca-wil-van-1-5-meterregel-af~b8700006/ ; Trouw: https://www.trouw.nl/economie/horeca-wil-van-anderhalvemeter-af-we-krijgen-het-niet-meer-uitgelegd~bc111546/ |
| 14-6-2020 | NL | News | The Netherlands pre-orders millions of vaccines that are under development in Oxford. | Volkskrant: https://www.volkskrant.nl/nieuws-achtergrond/nederland-heeft-miljoenen-doses-van-het-oxfordvaccin-besteld-hoe-kansrijk-is-het~bb87f3f8/; RTL nieuws: https://www.rtlnieuws.nl/nieuws/politiek/artikel/5150956/vaccin-corona-astrazeneca-farmaceut |
| 15-6-2020 | International | Safety measures | European Union loosens travel restrictions (within the EU). | NOS: https://nos.nl/artikel/2337304-nederlanders-mogen-weer-naar-buitenland-op-vakantie.html; Nu.nl: https://www.nu.nl/coronavirus/6055355/kabinet-versoepelt-op-15-juni-reisadvies-voor-europese-bestemmingen.html |
| 16-6-2020 | NL | News | The Dutch economy is expected to shrink by 6 percent this year, and unemployment is expected to double. | Volkskrant: https://www.volkskrant.nl/economie/economie-krimpt-dit-jaar-met-6-procent-en-werkloosheid-verdubbelt-verwacht-cpb~b15485b0/; NOS: https://nos.nl/artikel/2337409-cpb-ongekende-krimp-van-6-procent-werkloosheid-verdubbelt.html |
| 16-6-2020 | NL | News | An independent mid-term evaluation of the corona policy is announced. | AD: https://www.ad.nl/politiek/toch-onafhankelijk-onderzoek-naar-coronabeleid-kabinet~abafba3d/; Volkskrant: https://www.volkskrant.nl/nieuws-achtergrond/kamer-wil-toch-onafhankelijk-onderzoek-naar-coronabeleid~b33c364c/ |
| **16-6-2020** | **NL** | **Research** | **End data collection wave 4** |  |
| 17-6-2020 | NL | Statistics | The total number of patients that have tested positive has grown with 139 and the deaths have grown with 5. This brings the total number of cases to 49,087 and the number of people that died to 6,070. | WHO: https://covid19.who.int/region/euro/country/nl (retrieved on 11-08-2020) |
| 19-6-2020 | NL | News | Demonstration of the action group "Virus madness" is banned. | NOS: https://nos.nl/artikel/2337788-coronaprotest-in-den-haag-verboden-organisatoren-stappen-naar-rechter.html; Telegraaf: https://www.telegraaf.nl/nieuws/489283767/den-haag-verbiedt-anti-lockdownprotest-virus-waanzin-naar-de-rechter |
| 23-6-2020 | NL | News | First time since March 14 that no corona related deaths have been reported. | WHO: https://covid19.who.int/region/euro/country/nl (retrieved on 11-08-2020) |
| 24-6-2020 | NL | Safety measures | Government announces further loosening of corona measures. | NOS: https://nos.nl/artikel/2338386-kijk-hier-de-voorlopig-laatste-coronapersconferentie-terug.html; https://www.rijksoverheid.nl/onderwerpen/coronavirus-covid-19/vraag-en-antwoord/persconferentie-coronavirus-nieuwe-regels-per-1-juli-2020-in-eenvoudige-taal |
| 24-6-2020 | NL | Statistics | The total number of patients that have tested positive has grown with 64 and the deaths have grown with 5. This brings the total number of cases to 49,722 and the number of people that died to 6,095. | WHO: https://covid19.who.int/region/euro/country/nl |
| **24-6-2020** | **NL** | **Research** | **Start data collection wave 5** |  |
| 26-6-2020 | NL | News | Demonstration of the action group "Virus madness" is banned again. | NOS: https://nos.nl/artikel/2338608-den-haag-verbiedt-opnieuw-demonstratie-viruswaanzin.html; AD: https://www.ad.nl/binnenland/viruswaanzin-na-verbod-demonstratie-kom-morgen-niet-naar-malieveld~a93bb982/ |
| 30-6-2020 | NL | Statistics | The total number of patients that have tested positive has grown with 76 and the deaths have grown with 2. This brings the total number of cases to 50,223 and the number of people that died to 6,107. | WHO: https://covid19.who.int/region/euro/country/nl (retrieved on 11-08-2020) |
| **30-6-2020** | **NL** | **Research** | **End data collection wave 5** |  |

# References

Epskamp, S. (2020a). Psychometric network models from time-series and panel data. *Psychometrika*, *85*(1), 206-231. https://doi.org/10.1007/s11336-020-09697-3

Epskamp, S. (2020b). *Psychonetrics: Structural Equation Modeling and Confirmatory Network Analysis*. https://cran.r-project.org/web/packages/psychonetrics/index.html

Epskamp, S., Borsboom, D., & Fried, E. I. (2018). Estimating psychological networks and their accuracy: A tutorial paper. *Behavior Research Methods*, *50*(1), 195-212. https://doi.org/10.3758/s13428-017-0862-1

Epskamp, S., Cramer, A. O. J., Waldorp, L. J., Schmittmann, V. D., & Borsboom, D. (2012). qgraph: Network Visualizations of Relationships in Psychometric Data. *Journal of Statistical Software*, *48*(4), 1-18. http://www.jstatsoft.org/v48/i04/

R Core Team. (2013). *R: A language and environment for statistical computing*. R Foundation for Statistical Computing. http://www.R-project.org/

van Borkulo, C. D., van Bork, R., Boschloo, L., Kossakowski, J. L., Tio, P., Schoevers, R. A., Borsboom, D., & Waldorp, L. J. (2022). Comparing network structures on three aspects: A permutation test. *Psychological Methods*, *Advance online publication*. https://doi.org/10.1037/met0000476

1. The survey also contained items on smoking and alcohol consumption, but these items were excluded from further analysis due to the large amount of missing values. These items loaded on another component than *Healthy* *Lifestyle*. [↑](#footnote-ref-1)
2. We report the Huynh-Feldt corrected results given that epsilon was greater than .75. [↑](#footnote-ref-2)
